# Hydrogen in transport: a review of opportunities, challenges, and sustainability concerns

**DOI:** 10.1039/d5ra02918j

**Published:** 2025-07-10

**Authors:** Maha Awjan Alreshidi, Krishna Kumar Yadav, G. Shoba, Amel Gacem, S. Padmanabhan, S. Ganesan, L. Guganathan, Javed Khan Bhutto, P. Saravanan, Ahmed M. Fallatah, Muhammad A. Abo El-Khair, Jawaher Faisal Almalawi, Mir Waqas Alam, C. Kavitha, P. Tamizhdurai, A. Subramani

**Affiliations:** a Department of Chemistry, College of Science, University of Ha'il Ha'il 81441 Saudi Arabia; b Department of Environmental Science, Parul Institute of Applied Sciences, Parul University Vadodara Gujarat 391760 India; c Environmental and Atmospheric Sciences Research Group, Scientific Research Center, Al-Ayen University Nasiriyah Thi-Qar Iraq; d Department of Biotechnology, Dwaraka Doss Goverdhan Doss Vaishnav College (Autonomous) (Affiliated to the University of Madras, Chennai) 833, Gokul Bagh, E.V.R. Periyar Road, Arumbakkam Chennai 600 106 Tamil Nadu India; e Department of Physics, Faculty of Sciences, University 20 Août 1955 Skikda Algeria; f Department of Mechanical Engineering, Vel Tech Rangarajan Dr Sagunthala R&D Institute of Science and Technology Chennai Tamil Nadu India; g Department of Mechanical Engineering, Sathyabama Institute of Science and Technology Chennai Tamil Nadu India; h Department of Physics, Saveetha School of Engineering, Saveetha Institute of Medical and Technical Science (SIMATS) Thandalam Chennai-602105 Tamil Nadu India; i Department of Electrical Engineering, College of Engineering, King Khalid University Abha Saudi Arabia; j Department of Chemistry, St. Joseph's College of Engineering OMR Chennai-600119 Tamil Nadu India; k Department of Chemistry, College of Science, Taif University P.O. Box 11099 Taif 21944 Saudi Arabia; l Egyptian Petroleum Research Institute (EPRI) Nasr City Cairo 11727 Egypt; m Department of Biology, Adham University College, Umm Al-Qura University Makkah 21955 Saudi Arabia; n Department of Physics, College of Science, King Faisal University Al Ahsa 31982 Saudi Arabia; o Department of Chemistry, Dwaraka Doss Goverdhan Doss Vaishnav College (Autonomous) (Affiliated to the University of Madras, Chennai) 833, Gokul Bagh, E.V.R. Periyar Road, Arumbakkam Chennai 600 106 Tamil Nadu India tamizhvkt2010@gmail.com kavithakandhan@gmail.com; p Department of Biochemistry, Dwaraka Doss Goverdhan Doss Vaishnav College (Autonomous)Arumbakkam Chennai 600 106 Tamil Nadu India

## Abstract

This review provides a comprehensive and interdisciplinary assessment of the expanding role of hydrogen in enabling sustainable energy transitions within the transportation sector. Distinct from previous reviews, this study contributes original insights into advanced hydrogen storage technologies, integration across energy systems, and evolving policy frameworks. The review investigates the application of hydrogen in internal combustion engines, gas turbine propulsion, and fuel cell electric vehicles, with emphasis on recent progress in physical and chemical storage methods such as compressed gas, cryogenic liquid, metal hydrides, and sorbent based systems. It highlights innovations in material science and combined storage strategies that aim to improve safety, increase energy density, and reduce operational costs. Furthermore, the review explores the integration of hydrogen with renewable electricity and industrial systems, identifying its role in smart grid support, energy balancing, and flexible energy storage. The policy analysis highlights strategic national and international efforts that promote hydrogen deployment, including financial incentives, infrastructure development, and regulatory standards. The review also examines lifecycle assessment findings, comparing environmental impacts across hydrogen production routes and end use applications. Special attention is given to the role of alternative hydrogen carriers such as ammonia and liquid organic hydrogen compounds, which offer promising solutions for long distance transport and international trade. Finally, the study identifies current challenges related to technology standardization, safety regulations, and global market alignment, while outlining key research and innovation priorities essential to realizing a sustainable and inclusive hydrogen future for transportation.

## Abstract 2

This review provides a comprehensive and interdisciplinary assessment of the expanding role of hydrogen in enabling sustainable energy transitions within the transportation sector.

## Graphical abstract

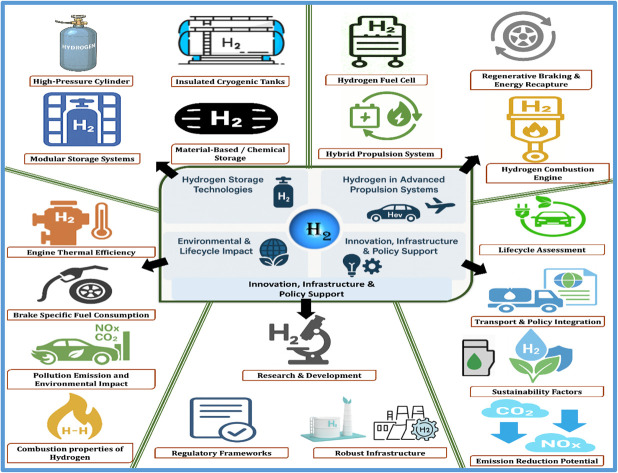

## 1. Introduction

The shift from fossil fuels to renewable energy sources is crucial in addressing climate change and achieving the united nations' sustainable development goals.^1^ The burning of fossil fuels emits greenhouse gases, notably carbon dioxide, which contributes significantly to global warming.^2^ This, in turn, results in a range of harmful environmental consequences, such as more frequent catastrophic weather occurrences, rising sea levels, and threats to both ecosystems and human health.^3^ Additionally, dependence on dwindling fossil fuel supplies heightens energy vulnerabilities, exacerbates geopolitical tensions, and fosters economic instability.^4,5^ Renewable energy sources such as hydro-electric, solar power, geothermal and wind energy present a cleaner and more sustainable alternative.^6^ They contribute to reducing environmental harm and support the achievement of lasting energy stability. These resources generate little to no emissions and are widely available across the globe.^7^ Adopting renewable energy reduces environmental damage, enhances energy independence, improves air quality and public health, and boosts the economy by creating jobs in growing green industries.^8^ To address the climate crisis and ensure a fairer, more stable future, it's crucial that the global community accelerates the shift to renewable energy.^9^ This transition is key to building stronger, more resilient societies and protecting the planet for future generations.^10^

Shifting from fossil fuels to renewable energy sources represents a key approach to tackling the global climate crisis and fulfilling the targets set by the united nations sustainable development goals.^11^ Fossil fuel combustion is a major contributor to human-induced greenhouse gas emissions, which drive the rise in global temperatures, intensifying extreme weather events, accelerating sea-level rise, and posing severe threats to ecological integrity and human well-being.^12,13^ Moreover, the depletion of fossil fuel reserves exacerbates concerns over energy security and heightens geopolitical tensions.^14^ Conversely, renewable energy systems such as hydro-electric, solar power, geothermal and wind energy represent environmentally sustainable alternatives that contribute to improved ecological quality, enhanced energy system stability, and socioeconomic advancement through employment opportunities.^15,16^ A global–scale transition to these resources is essential for fostering a sustainable, low-carbon, and climate-resilient future.^17^ Within this context, hydrogen emerges as a particularly promising energy carrier due to its abundance, high specific energy (∼120 kJ g^−1^ LHV), and environmentally benign combustion, which produces only water vapor rather than carbon dioxide.^18^ These attributes position hydrogen as a critical component in reducing greenhouse gas emissions and supporting a diversified, decarbonized energy portfolio.^19,20^ It offers a sustainable way to reduce GHG emissions and improve air quality.^21^ Versatile in use, hydrogen can power vehicles, generate electricity, and support industrial processes like oil refining.^22,23^ Furthermore, the flexibility of renewable technologies allows for energy production from a diverse array of natural sources, positioning them as essential drivers in the global shift away from fossil fuel dependence.^24^

**Fig. 1:**
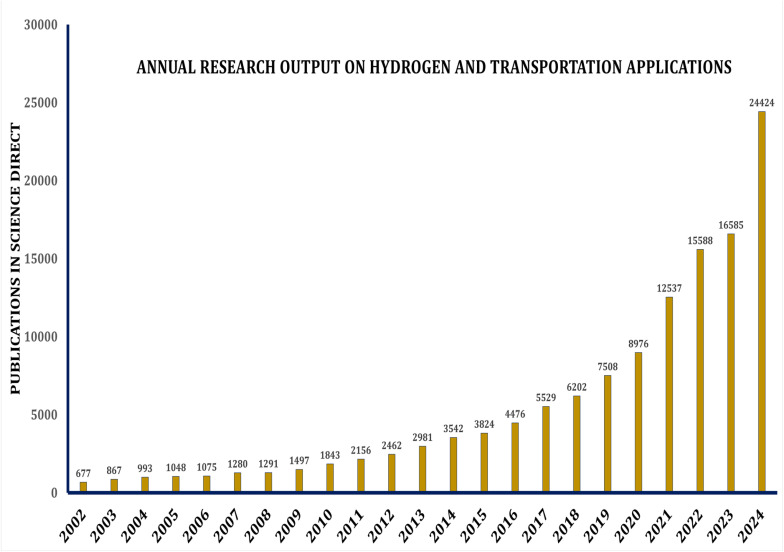
Annual research output on hydrogen and transportation applications. (Data were retrieved using the Scopus and science direct databases by applying the boolean keyword combination: (“hydrogen” and (“transportation” or “fuel cell” or “automotive” or “aviation”)). The selection scope included peer-reviewed journal articles published between 2002 and 2024).

Hydrogen is gaining momentum as a versatile, low-emission energy source, with growing applications across sectors like transportation and manufacturing. In the transportation sector, hydrogen presents a low-emission alternative to conventional fossil fuels.^25^ Hydrogen fuel cell technology is currently employed in various types of vehicles, while ongoing research and development efforts are investigating its potential application in aviation, including the development of hydrogen-powered aircraft.^26^ Research interest has surged, as shown in Fig. 1 which charts Scopus-indexed publications with “hydrogen” and transport-related terms in the title. Since 2020, studies have grown rapidly, averaging 32% annual growth, with a peak increase of about 65% in 2021.^27^

Although hydrogen offers significant advantages as a clean fuel, several challenges must still be addressed before it can realize its full potential. One of the main challenges lies in the expenses related to the production and storage of renewable energy.^28^ Currently, the most used technique for producing hydrogen is steam methane reforming (SMR). However, this process is energy-intensive and produces carbon dioxide as a by-product. More efficient and sustainable methods, such as electrolysis and biological processes, have been developed but are not yet as widely adopted.^29^ Additionally, the infrastructure for hydrogen refueling remains limited. While the number of hydrogen fueling stations is growing, it is still far behind the number of conventional gas stations, decreasing consumption for hydrogen energy and delaying the uptake of hydrogen-powered fuel cell automobiles.^30^

Ongoing ventures into research and development, coupled with the growth of enabling infrastructure, is positioning hydrogen as a fundamental component of the global shift toward a sustainable energy future.^31^ This review explores the prospect of hydrogen as a fuel for transit, focusing specifically on its role in the automotive and aviation sectors. The article is structured into nine sections: an introduction and overview of the methodology, followed by an exploration of hydrogen storage and safety considerations (Section 3). Sections 4 and 5 delve into the use of hydrogen in internal combustion engines and turbine-based propulsion systems, respectively. Section 6 focuses on electric vehicles powered by hydrogen fuel cells, while Section 7 presents a comprehensive evaluation of environmental and energy performance throughout the product's lifespan of hydrogen's environmental and energy impacts. Future prospects and challenges are discussed in Section 8, and the review concludes in Section 9 with a synthesis of key insights and recommendations.^32–34^ Hydrogen is emerging as a critical clean energy solution to address global climate goals, especially in transportation. Its high energy content and eco-friendly combustion make it a strong candidate to replace fossil fuels.

## 2. Research strategy

This review presents a comprehensive and multidimensional analysis of hydrogen as a flexible energy vector within the transportation sector, with a particular focus on its deployment in combustion-based engines, turbine driven propulsion systems, and hydrogen powered fuel cell vehicles. The study is underpinned by a structured and transparent methodology designed to ensure both thoroughness and reproducibility in the evaluation of technical advancements, storage innovations, and policy frameworks. The literature review was conducted using a methodical, phased approach. It began with the identification of recent and relevant research publications to reflect emerging trends and state of the art developments in hydrogen storage and application technologies.^35^ Subsequently, earlier foundational studies were incorporated to capture the evolution and historical context of hydrogen research. The time frame for literature selection spanned from 2010 to 2024, encompassing both foundational knowledge and the latest progress in hydrogen related science and policy.

Peer reviewed journal articles, review papers, technical reports, and policy documents from reputable international organizations were considered for inclusion. Databases consulted included Science Direct, Scopus, Springer Link, Wiley Online Library, and Google Scholar, ensuring interdisciplinary coverage across engineering, energy, and regulatory domains. The search strategy employed targeted keywords and Boolean combinations such as “hydrogen storage technologies”, “fuel cell electric vehicles”, “hydrogen internal combustion engines”, “gas turbine hydrogen combustion”, “hydrogen transport infrastructure”, “hydrogen energy policy”, and “hydrogen lifecycle assessment”. Supplementary terms such as “green hydrogen”, “hydrogen integration with renewables”, and “hydrogen refueling station” were also used. To preserve the scientific rigor and relevance of the review, strict inclusion criteria were followed: (1) peer reviewed research and systematic reviews, (2) studies presenting experimental results, technoeconomic models, or lifecycle assessments, and (3) policy documents issued by government agencies or globally recognized institutions. Exclusion criteria included: (1) non-peer-reviewed or non-academic literature such as white papers, editorials, and blogs, (2) documents not available in English, (3) duplicate content across databases, and (4) publications focused exclusively on unproven or limited scale hydrogen production methods such as microbial electrolysis without field validation. This systematic search resulted in the selection of over 220 primary sources, which were then categorized thematically across key focus areas: hydrogen storage and distribution technologies, propulsion systems, emission characteristics, safety analysis, and supportive policy frameworks.

The review further evaluates a range of hydrogen storage solutions—including physical methods such as compressed and cryogenic storage, and chemical options including metal hydrides and liquid carriers—while addressing critical factors like system safety, volumetric efficiency, and distribution logistics. In propulsion applications, core performance metrics such as combustion characteristics, thermal efficiency, fuel economy, and emissions output are analyzed. For gas turbines, the study places particular emphasis on hydrogen's combustion behavior and fuel adaptability. In the context of Fuel Cell Electric Vehicles (FCEVs), the environmental impact and energy efficiency are assessed through lifecycle analysis (LCA), providing insights into total energy consumption, sustainability, and carbon emissions.

**Fig. 2:**
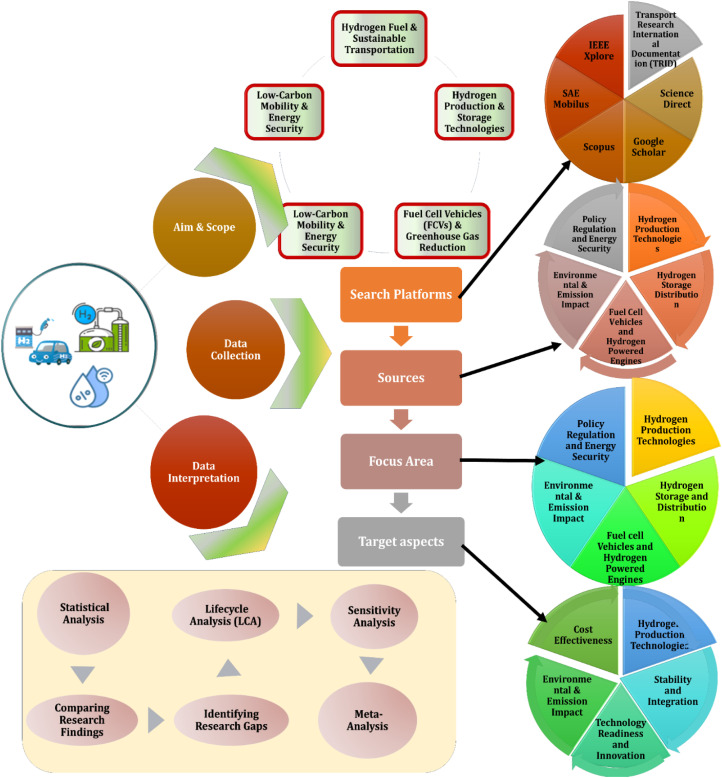
Flowchart diagram of methodological steps.

The overall methodology is organized into three major phases: defining the research scope, sourcing and screening relevant literature, and interpreting findings across technical, environmental, economic, social, and safety related dimensions. Fig. 2 presents a visual representation of this methodology, outlining the step-by-step flow from defining objectives to data analysis.^36,37^ The literature review was carried out using a structured and methodical approach, beginning with the most current and relevant publications to capture recent advancements and emerging trends in the field.^38^ The initial phase involved targeted searches in academic databases and platforms such as Google Scholar and Science Direct, employing specific keywords associated with hydrogen storage, combustion-based engines, turbine-driven propulsion systems, and hydrogen-powered fuel cell vehicles.^39^ At this stage, emphasis was placed on recent peer-reviewed journal articles, in-depth review papers, and detailed technical reports.^40^ Following this, the scope of the review was extended to include earlier studies, allowing for a thorough understanding of the historical development and progression of research within this domain.^41^ A structured literature review spanning 2010–2024 was conducted to assess hydrogen storage, vehicle integration, and policy evolution. The approach ensures a balanced, interdisciplinary understanding.

## 3. Ensuring safe and efficient hydrogen storage

**Fig. 3:**
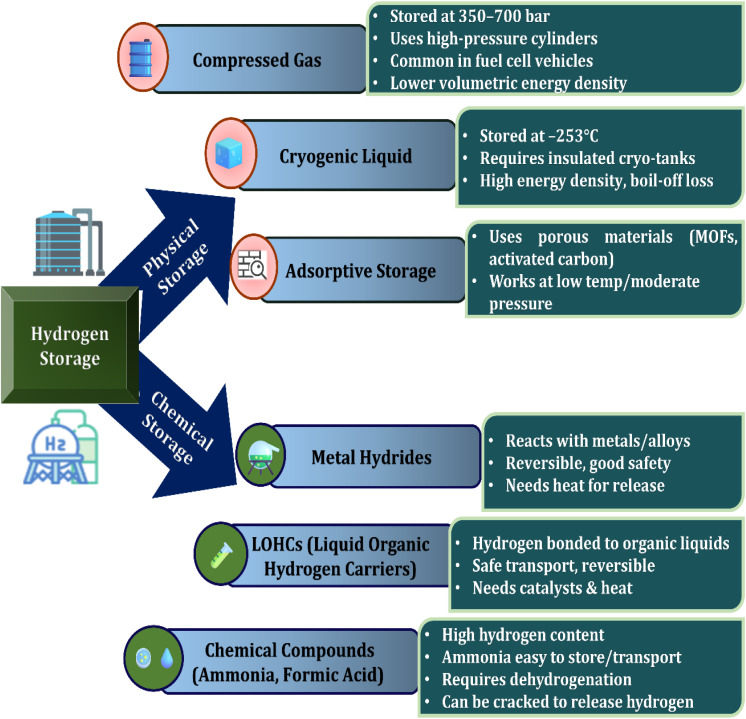
Hydrogen storage: methods and classifications.

Hydrogen storage represents a fundamental consideration in assessing its viability as an energy carrier, as demonstrated in Fig. 3. This section explores the current physical and chemical hydrogen storage technologies, analyzing their efficiency, safety, and practicality for transportation. A range of storage methods is currently employed, including chemical storage, compressed gas, and liquid hydrogen.^42^ Each of these approaches offers distinct advantages and presents specific challenges, particularly in terms of safety, cost-effectiveness, and energy density.^43^ The unique physical and chemical properties of hydrogen pose considerable technical obstacles to its storage and distribution, necessitating ongoing research and innovation to develop efficient and scalable solutions.^44^ Compressed gas storage is prevalent because of its relatively simple infrastructure, but it involves very high pressure levels, leading to potential safety risks.^45^ Liquid hydrogen, on the other hand, offers improved energy density but demands advanced cryogenic systems to maintain ultra-low temperatures.^46^ Chemical storage stands out as a potentially safer method, though it typically requires extra energy to extract usable hydrogen. Looking ahead, it is essential to invest in research aimed at refining these storage approaches to achieve greater efficiency, improved safety standards, and reduced costs. Innovations in material science may lead to more practical chemical storage solutions, while integrated or hybrid systems combinng multiple technologies could enhance overall system efficacy and reliability.^47,48^

### 3.1. Hydrogen storage technologies: a physical approach

A physical approach – introduces methods like compressed and cryogenic hydrogen storage and evaluates their technical and safety characteristics.

#### 3.1.1. Compressed hydrogen: methods and technologies for storage

Compressed hydrogen storage is among the most commonly employed methods due to its relative simplicity and cost-effectiveness. This approach typically involves storing hydrogen at pressures lies between 350 to 700 bar (approximately 5000 to 10 000 psi) within cylinders fabricated from steel, aluminum, or advanced composite materials.^49^ Despite its widespread use, high-pressure hydrogen storage introduces challenges related to mechanical stress, which may lead to material fatigue and potential structural failure. To address these concerns, especially the risk of hydrogen-induced embrittlement, the use of robust and chemically resistant materials is indispensable.^50^ Storing vessels are generally categorised into 4 groups: Type I consists entirely of metal; Type II incorporates a metal liner reinforced with a fiber based resin composites; the Type III employs a metal liner encased in a composite shell; and Type IV consists of a carbon fibre composite structure with a polymer liner. Although Type IV tanks are noted for their efficiency and lightweight properties, high-pressure storage systems continue to pose limitations in terms of volume and safety, particularly when applied in mobile or transportation contexts.^51,52^ Hydrogen can be stored using physical (compressed gas, cryogenic) or chemical (metal hydrides, sorbents) methods. Ongoing innovation is crucial to improve safety, density, and scalability for transport applications.

#### 3.1.2. Exploring cryogenic and liquid hydrogen for transportation and energy

**Fig. 4:**
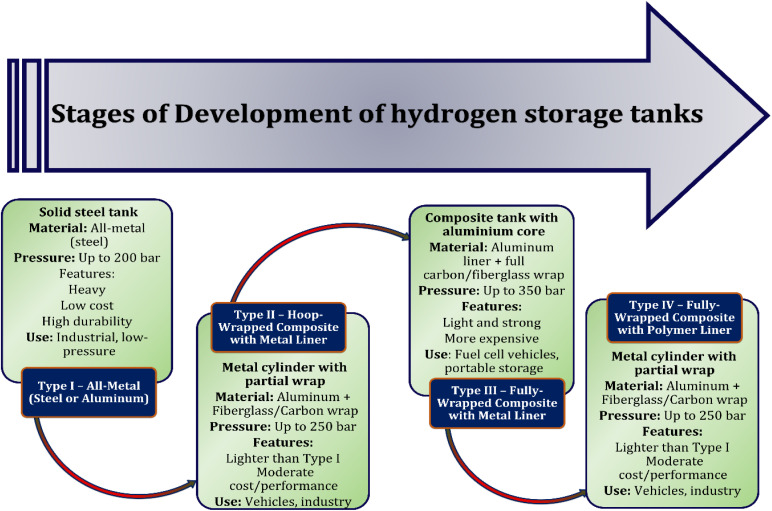
Different configurations of hydrogen storage tanks.

Cryogenic storage of hydrogen involves cooling the gas to temperatures below minus 253 degrees Celsius, thereby transforming it into a liquid to achieve a significantly enhanced energy intensity.^53^ Applications for this technique are especially common in the aviation sector, where the ability to store substantial amounts of energy within a limited volume is a critical requirement.^54^ However, maintaining hydrogen in its liquid state necessitates the use of advanced, vacuum-insulated storage systems, commonly constructed from materials such as aluminum or steel.^55^ These tanks are complex and expensive to produce due to the stringent thermal insulation and structural integrity required to handle such extremely low temperatures. To address some of the limitations, alternative methods are under investigation.^56^ These include cryo-adsorption, high-pressure cryo-compression (up to 800 bar), and improved liquefaction technologies. Cryo-compression, in particular, helps limit hydrogen boil-off but comes with the need for sophisticated equipment and precise temperature–pressure control. Future advancements should concentrate on enhancing thermal insulation, minimizing energy consumption during storage and transfer, and developing robust infrastructure for refuelling especially in commercial transport applications. As illustrated in Fig. 4 various hydrogen storage cylinders are under development, each designed to balance safety, efficiency, and performance.^57^ While cryogenic storage offers high energy density, it still presents significant financial and technical obstacles that need to be removed in order to be adopted more widely.

### 3.2. Hydrogen storage *via* chemical methods

Chemical repository strategies such as sorbent materials, metal hydrides, and chemical hydrides exist as a viable substitute for conventional hydrogen repository methods due to their ability to achieve higher volumetric energy densities.^58^ Metal hydrides, including those based on magnesium and lithium, can absorb hydrogen under moderate conditions, but practical deployment is hindered by slow hydrogen release rates and material cost. Chemical hydrides store hydrogen in a dense form, though their use often depends on catalysts to facilitate hydrogen liberation.^59^ Sorbents like metal–organic frameworks (MOFs) and zeolites offer reversible hydrogen uptake, yet they still require significant improvements in storage capacity and operational performance. To advance these technologies, future efforts should prioritize the development of lightweight, economical materials that release hydrogen more rapidly. Combining different storage mechanisms includes integrating metal hydrides with sorbents, could yield systems with enhanced safety, efficiency, and scalability. Additionally, breakthroughs in catalyst design and nanomaterial engineering may accelerate the feasibility of storing hydrogen for use in wide range of energy sectors.^60–62^

### 3.3. Hydrogen fueling the future of transportation

**Fig. 5:**
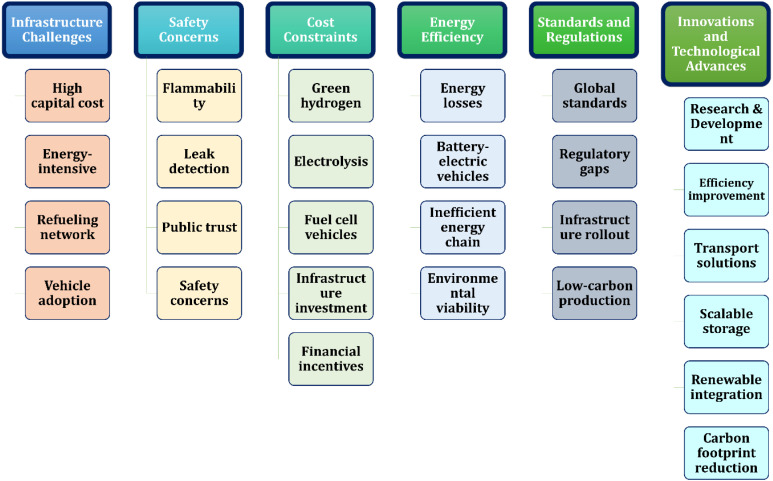
Sustainability constraints in hydrogen development.

The effective transportation of hydrogen from production facilities to end-users is essential for its acceptance as a workable substitute for traditional fossil fuels.^63^ High-pressure tubular trailers, cryogenic fluid tanks, and customized pipes are examples of common approaches to transit. However, several technical and logistical challenges complicate the large-scale distribution of hydrogen, as illustrated in Fig. 5. Due to its high flammability and low molecular weight, hydrogen cannot be safely or efficiently transported using standard gas distribution infrastructure.^64^ Although it possesses a high energy content by weight, hydrogen requires specialized containment systems to ensure safe handling and delivery. To enable widespread hydrogen use, several key issues must be addressed. These include the development of standardized transportation protocols, enhanced safety measures, improved economic viability, increased public engagement and awareness, and the implementation of supportive regulatory frameworks. Addressing these factors will be crucial in positioning hydrogen as a sustainable and broadly utilized energy solution.^65–67^

**Fig. 6:**
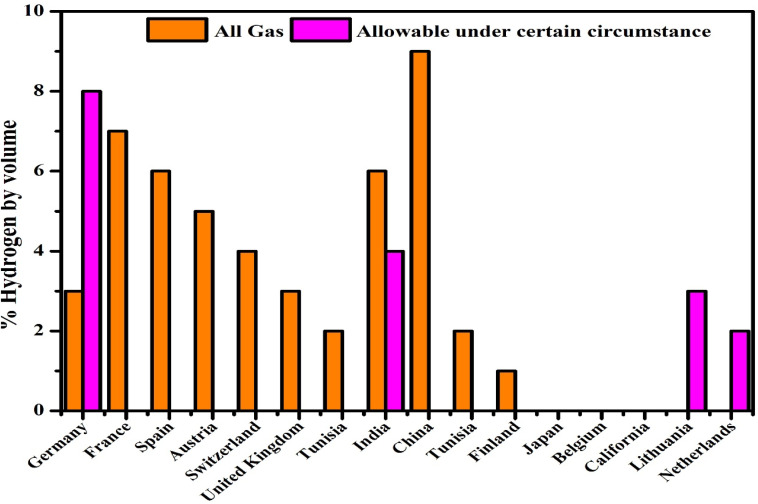
National regulations on hydrogen proportions in natural gas supply data extracted from ref. 72 using open-source regulatory documentation and cross-validated with policy repositories from IEA and national energy agencies. Search criteria included keywords: “hydrogen blending”, “natural gas pipelines”, “regulations”, “percentage limits”, “country comparison”.

Transporting hydrogen *via* pipelines poses safety and cost challenges. Due to its low density, hydrogen needs high-pressure transport and occupies more space, increasing expenses. Leakage risks also raise explosion concerns since hydrogen reacts easily with oxygen.^68,69^ Despite this, safe pipeline transport is possible using pressure valves, leak sensors, and fire prevention systems. Bai and co-researchers^70^ conducted a comprehensive review of six distinct hydrogen delivery pathways. These include transportation *via* gaseous hydrogen in tube trailers, distribution through dedicated hydrogen pipelines, liquid hydrogen transport using specialized trucks, the incorporation of hydrogen into the current networks of natural gas networks, as well as the use of chemical carriers such as liquid ammonia and methanol. Among these, short-range pipelines (up to 1800 km) were found to be the most cost-effective. Long-range pipelines had lower transport costs but required high capital investment.^71^ As illustrated in Fig. 6. Germany permits a hydrogen blend of up to 10%, representing the highest allowable percentage among the compared countries. Blending hydrogen into NG pipelines is another option. Operational difficulties are usually modest when hydrogen is mixed with natural gas at concentrations lower than about 5 to 15 percent. Blends of 15–50% may need equipment upgrades, while over 50% raises major safety and material concerns. Germany currently permits up to 10% hydrogen blending. Studies suggest 15–20% is generally safe.^73^

Containers such as compressed gas, cryogenic tanks, and metal hydride systems—offer an alternative to pipelines but are only suitable for short-distance hydrogen transport. For sustainability, a resource must be viable for both import and export. Hydrogen's flammability makes transport difficult. Liquid hydrogen tankers can be used for export, but they are costly and require specialized equipment.^74^ Truck transport is currently the cheapest option. Overall, hydrogen export is still emerging, with many technical and economic challenges. However, ongoing innovation is expected as demand for clean energy grows.^75^

## 4. Hydrogen-powered internal combustion engines: a sustainable future

**Fig. 7:**
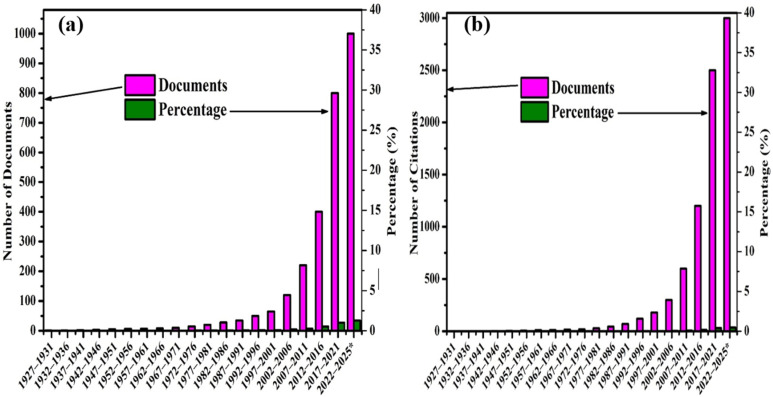
Trends in scientific research on hydrogen-fueled internal combustion engines (H_2_ICEs) from 1925 to 2023: (a) annual number of publications, and (b) annual number of citations.

Hydrogen use in internal combustion engines (ICEs) has gained significant attention for its potential to lower emissions without sacrificing performance. Leveraging existing ICE infrastructure through retrofitting or dual-fuel systems offers a practical path toward cleaner energy. This part delves into hydrogen's application in internal combustion engines, focusing on injection strategies, performance metrics, emissions, and environmental benefits. As oil reserves decline and environmental regulations tighten, hydrogen stands out as a renewable fuel due to its fast flame speed, high energy content, and non-toxic combustion products. Interest in hydrogen-powered ICEs is growing, as shown by the rise in related publications (Fig. 7). Future research should aim to optimize engine designs for improved hydrogen combustion and efficiency.^76,77^

**Fig. 8:**
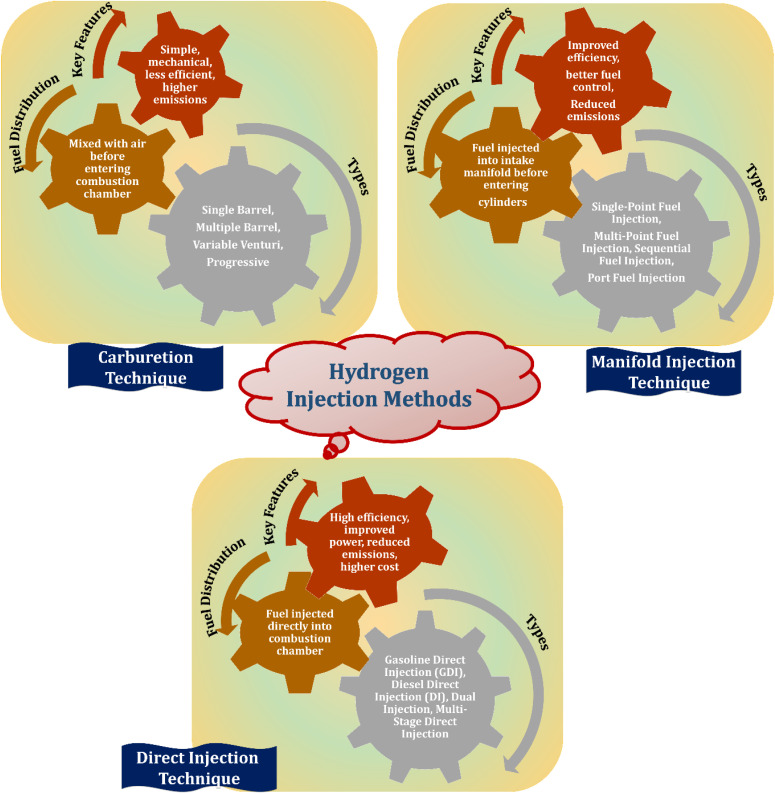
Hydrogen injection methods for energy and transport systems.

Fig. 8 illustrates the different hydrogen injection methods. In the case of direct injection, hydrogen is delivered directly into the engine's combustion chambers, where it mixes with air and ignites to produce power. Hydrogen shows strong potential as an accompanying fuel in diesel IC engines. Its high diffusivity allows better air-fuel mixing and efficient combustion. Researchers have explored hydrogen as a secondary fuel to reduce reliance on hydrocarbons. Due to its high self-ignition temperature, hydrogen can be used in compression ignition engines with assistance from spark or glow plugs. Mixing hydrogen with diesel shortens combustion duration because of its faster flame speed.^78,79^ Hydrogen can be infused using either the direct infusion or terminal fuel infusion. Port injection, where hydrogen enters the intake manifold before air, is preferred for its simplicity. In direct injection, hydrogen is delivered directly into the engine cylinders. Fig. 8 illustrates the different hydrogen injection methods.^80^

**Fig. 9:**
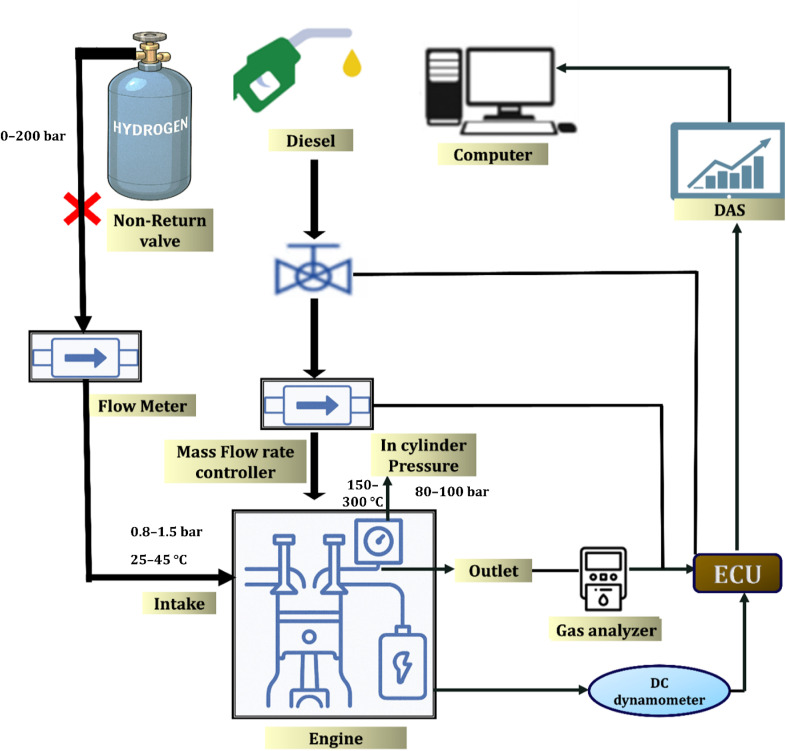
Experimental configuration of a diesel engine enhanced with hydrogen.

Inlet manifold injection is considered a cost-effective method for introducing hydrogen into internal combustion engines. However, its performance is constrained by various engine operating parameters. This technique is frequently associated with issues such as backfire, knocking, and pre-ignition, which are primarily caused by inadequate ignition energy. To mitigate these risks and prevent potential engine damage, flashback arrestors are commonly employed.^81,82^ Hydrogen's short quenching distance increases knock risk, regardless of engine speed. It also restricts intake airflow, causing oxygen shortages and lowering volumetric efficiency. To address this, hydrogen flow is adjusted based on engine settings. As shown in Fig. 9 up to 40% hydrogen can be used at medium load, but it must be reduced to 25% at high torque and speed. Optimizing hydrogen ratio and engine load enhances thermal efficiency and enables knock-free operation. Direct injection is recommended to eliminate knock and backfire.^83,84^ The setup used a single-cylinder, four-stroke diesel engine (model: Kirloskar TV1) coupled to an eddy current dynamometer for load application. Hydrogen was introduced using a Bosch GDI injector rated at 20 MPa. Intake air pressure was maintained at atmospheric level, and hydrogen pressure was regulated *via* a boost pump. Combustion parameters were monitored using AVL combustion analyzer with in-cylinder pressure transducer (Kistler 6056A). Engine tests were conducted at varying loads (25%, 50%, 75%, 100%) under ambient temperature (∼27 °C) and controlled humidity. Injection timing and pressure were adjusted to prevent knocking and optimize performance.

Direct injection of hydrogen into internal combustion engines offers notable advantages, including the reduction of volumetric efficiency losses and the facilitation of mixture stratification, which together contribute to increased power output.^85,86^ Furthermore, precise control over injection timing plays a crucial role in mitigating the risk of engine knocking. Compared to other renewable gaseous fuels such as natural gas, bioethanol, and ammonia, hydrogen demonstrates superior thermal efficiency, highlighting its potential as a high-performance alternative.^87^ In a recent study, Liu and collaborators^88^ introduced hydrogen directly into the piston bowl using a modified gasoline direct injection system. This injector was installed through the glow plug port and strategically positioned adjacent to the diesel pilot injector to enhance combustion characteristics. The GDI (Bosch) was chosen for its sealing performance, with no injector failures reported. A drop of oil was added before each test for lubrication. Hydrogen was injected at 20 MPa using a boost pump, and pressure rise was measured using the Zeuch method.^89^

Advanced ignition systems help prevent flame blowout in combustion chambers. Among the advanced ignition techniques under investigation, laser-assisted and plasma-assisted ignition systems have garnered considerable interest due to their potential to enhance combustion efficiency and overall engine performance. In a recent study, Yilmaz and colleagues^90^ employed a Q-switched laser to initiate combustion in methane–hydrogen fuel mixtures, demonstrating the viability of this method in improving ignition reliability and fuel utilization. Their results showed laser ignition offers more precise and controllable combustion than traditional spark systems, especially under linear conditions, leading to lower emissions and fuel consumption. Hydrogen addition increased flame speed, resulting in a more wrinkled flame front. However, laser systems may pose safety risks like flashback and detonation. Zhao and collaborators^91^ investigated plasma ignition using a zero-dimensional simulation model that integrated SENKIN with ZDplaskin. Their findings revealed that plasma ignition not only achieved faster ignition compared to conventional spark ignition but also required just one-tenth of the energy input. This improved performance was attributed to the formation of highly reactive radicals, such as atomic oxygen, hydrogen, and hydroxyl, which were produced from the interaction between ammonia and oxygen. The presence of these species significantly enhanced the combustion process, underscoring the potential of plasma ignition as an energy-efficient and effective alternative for initiating fuel combustion.^92^

### 4.1. Engine thermal efficiency

#### 4.1.1. Assessing brake thermal efficiency in powertrains

The air-to-fuel ratio optimization according to engine load conditions plays a critical role in influencing brake thermal efficiency (BTE). Key contributing factors include the calorific value of the fuel and the quality of atomization. Hydrogen, due to its superior diffusion and combustion characteristics, facilitates improved atomization, thereby enhancing BTE.^93^ Although variations in injection rate generally have minimal impact on BTE, adjustments in injection timing—particularly advancing it—can slightly reduce BTE under conditions of elevated cylinder pressure. In general, hydrogen-enriched fuel blends tend to yield higher BTE, attributed to their elevated flame speed and improved effectiveness in heating. It has been established by Kanth *et al.*^94^ that supplying hydrogen at a flow rate of 7 litters per minute led to an increase in BTE by 3.32% for a blend containing 10% biodiesel and by 1.92% for a 20% biodiesel blend. Furthermore, while increased injection pressure improved BTE in diesel-fuelled engines, it resulted in a decline in BTE for biodiesel blends when pressures exceeded 240 bar. Enhanced injection timing was found to be beneficial for biodiesel combustion, contributing to higher thermal efficiency through more complete fuel oxidation. In a complementary study, Seelam *et al.*^95^ reported that introducing hydrogen at a rate of 12 litters per minute under 75% engine load conditions led to a 6.77% increase in peak cylinder pressure and a 1.50% reduction in the heat release rate compared to diesel operation. Additionally, emissions of hydrocarbons and nitrogen oxides were reduced by 6.66% and 10%, respectively, while thermal efficiency improved by 5%. These improvements are primarily attributed to hydrogen's catalytic effect on combustion, encouraging the possibility of a more consistent fuel-air combination, which in turn facilitates faster combustion rates and a higher proportion of premixed combustion.^96^

#### 4.1.2. Brake specific fuel consumption analysis (BSFC)

**Fig. 10:**
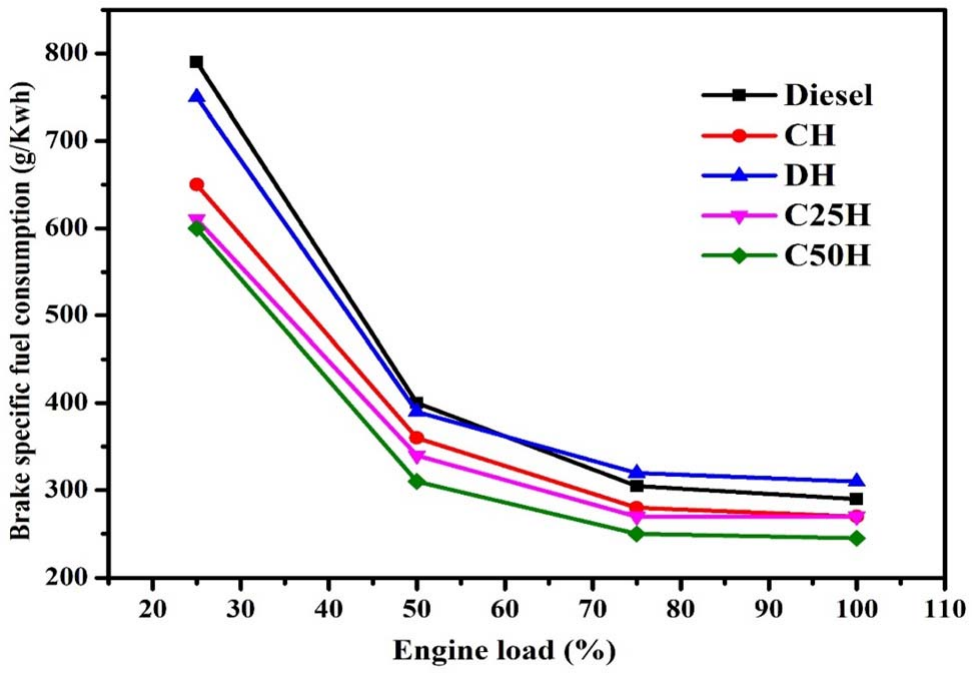
Impact of hydrogen on brake specific fuel consumption (BSFC).^97^

Incorporating hydrogen into the engine reduces BSFC across various loads by increasing the calorific value in the combustion chamber, improving BTE, and lowering fuel consumption. Hydrogen's high flammability, fast flame speed, short quenching distance, and low viscosity allow for more efficient combustion, enabling the engine to use less diesel. Regardless of braking power or engine load, the additional calorific value lowers fuel consumption. As BSFC and heating value are linked, increasing hydrogen intake lowers diesel consumption at all brake power levels, as shown in Fig. 10.

#### 4.1.3. Energy consumption per brake power output

The integration of hydrogen in diesel engines has been shown to reduce Brake Specific Energy Consumption (BSEC), primarily due to hydrogen's high energy content relative to conventional diesel fuel. Owing to its higher calorific value, hydrogen contributes to lower overall energy consumption during engine operation. As the proportion of hydrogen in the fuel blend increases, a gradual reduction in BSEC is typically observed.^98^ Seelam *et al.*^99^ explored that a blend consisting of 70% diesel and 30% 1-pentanol (referred to as D70P30), when injected at 15° before top dead center (bTDC), resulted in a 7.21% increase in BSEC under full load conditions. However, the inclusion of hydrogen to this blend leads to a 6% reduction in BSEC compared to the D70P30 blend alone. This inverse relationship is attributed to hydrogen's rapid combustion characteristics and its narrower quenching distance relative to diesel, which together promote more efficient energy release and improved combustion dynamics.

#### 4.1.4. Power generation and efficiency metrics

In the combustion chamber, the hydrogen energy fraction affects the coefficient of variation and the indicated mean effective pressure, or IMEP. Below a 50% hydrogen ratio, IMEP remains stable, but it increases as the hydrogen fraction rises, peaking at 80%. At this point, the efficiency reaches an impressive 50.6%, far surpassing diesel. Similarly, the coefficient of variance decreases as the hydrogen ratio increases.

### 4.2. Combustion properties of hydrogen fuel

Injection timing has a significant impact on the dynamics of in-cylinder pressure in diesel engines. Advancing the injection timing typically leads to a hike in peak pressure and a more rapid pressure rise, primarily due to a prolonged ignition delay that allows for improved fuel-air mixing. In contrast, retarded injection timing yields a more gradual pressure increase. The peak cylinder pressure and maximum combustion temperature are influenced by a combination of injection timing, fuel characteristics, and engine operating conditions.^100^ By modifying injection timing, the pressure peak should ideally occur shortly thereafter the top dead-center (TDC), engine designers can effectively manage combustion temperature and pressure levels. Advanced injection timing initiates combustion earlier, leading to a longer delay in ignition and increased HRR during the premixed condition. However, excessive advancement may diminish combustion efficiency. Additionally, increasing the quantity of injected fuel raises the in-cylinder pressure and accelerates combustion. When hydrogen is blended with biodiesel, a notable increase in cylinder pressure is often observed. This gain is related to better the atomization process and the ignition process interruption, which can be further tuned through improving spray penetration. Ignition delay is sensitive to in-cylinder temperature and engine load, with higher loads typically resulting in lower injection timings, especially given hydrogen's relatively high auto-ignition temperature.^101^ In hydrogen-biodiesel mixtures, ignition delay can be extended due to hydrogen's combustion properties, posing challenges for engine startup. Cylinder pressure significantly affects both the ignition and combustion processes.

Hydrogen-enriched fuels generally lead to higher cylinder pressures due to their elevated laminar flame speeds. Under high stoichiometric conditions, hydrogen combusts more rapidly than at lower hydrogen-to-air ratios, particularly at reduced engine loads. The heat release rate (HRR), an indicator of combustion duration, is governed by several fuel properties, including calorific value, viscosity, latent heat of vaporization, and cetane number.^102^ Fuels with higher viscosity introduce greater fuel mass into the cylinder, necessitating increased injection pressure to achieve finer atomization. This enhances HRR through improved combustion efficiency. HRR also depends on the balance between premixed and diffusion combustion phases. In diesel engines, increasing hydrogen content promotes leaner mixtures and higher HRR.^103^ However, excessive hydrogen concentrations can lower HRR due to hydrogen's faster burn rate compared to diesel, with most combustion occurring during the premixed phase. Delayed ignition can result in incomplete combustion, but enhanced atomization, which produces finer fuel droplets, helps maintain higher HRR and cylinder pressure than pure diesel. These fine droplets combust more efficiently, shortening the overall combustion duration. The early start of ignition (SOI) associated with hydrogen can be mitigated by delaying SOI, which reduces engine knocking and acoustic emissions while moderating HRR.^104^

As the proportion of hydrogen increases, the HRR tends to broaden and decrease in magnitude, indicating a less homogeneous hydrogen-to-air mixture. Higher hydrogen content reduces knocking but increases peak pressure, enabling modulation of flame speed through premixed combustion strategies. Liu *et al.*^105^ emphasized that transitioning from conventional to premixed hydrogen combustion can intensify peak pressure and HRR due to the resulting imbalance. The high flame propagation speed of hydrogen shortens combustion duration relative to conventional biodiesel blends and diesel. Kanth *et al.*^106^ observed no consistent relationship between injection timing, injection pressure, and hydrogen fuel consumption. Moreover, elevated injection pressures have been found to reduce cylinder temperatures and restrict flame propagation, as corroborated by Liu *et al.*^107^ and Seelam *et al.*^108^

### 4.3. Pollutant emissions and environmental impact

#### 4.3.1. CO_2_ emission trends and analysis

**Fig. 11:**
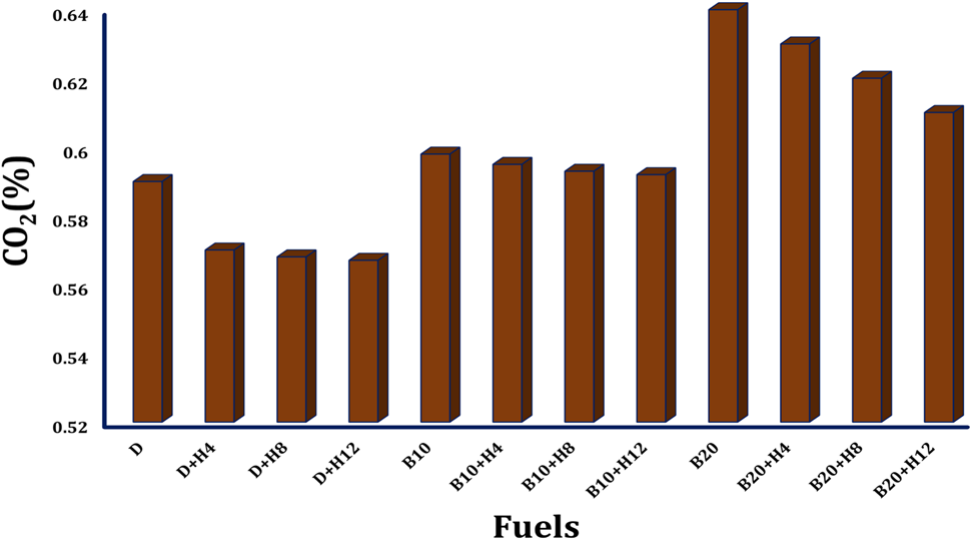
Comparison of carbon dioxide emissions across various fuel type.^109^

The usage of hydrogen based diesel engines offers a promising strategy for significantly reducing carbon dioxide emissions, primarily because hydrogen contains no carbon. As the concentration of hydrogen in the fuel mixture increases, the combustion temperature also rises. This enhances thermal efficiency and improves spray uniformity due to the high flame speed of hydrogen. As a result, carbon dioxide emissions decrease substantially, from 643 grams per kilowatt hour to 142 grams per kilowatt hour, as shown in Fig. 11. This notable reduction is mainly due to more complete combustion, which promotes the conversion of carbon monoxide into carbon dioxide.

#### 4.3.2. NO_*x*_ emissions in transportation and industry

When hydrogen is added to a diesel engine, more nitrogen oxides are produced. Because of its high autoignition temperature, hydrogen causes increased NO_*x*_ and cylinder temperatures. Increasing the air-to-fuel ratio speeds up combustion, which lowers NO_*x*_ production. Better fuel combustion results in higher NO_*x*_ emissions. Furthermore, more NO_*x*_ was produced as a result of improved injection timing. One of the biggest obstacles to present research is the lowering of NO_*x*_. The production of NO_*x*_ can be reduced by lowering the calorific value of the fuel.^110^ Conversely, the BTE and BP will significantly decline. For many years, there has been discussion on how to balance reduced NO_*x*_ output with increased efficiency. The long-term sustainability of fuels derived from hydrogen in the energy sector remains a subject of debate, as nitrogen oxide emissions continue to pose a significant challenge. Although advanced technologies such as exhaust gas recirculation, selective catalytic reduction, and the usage of nanoparticles have shown potential in mitigating nitrogen oxide levels to some degree, the emissions produced are still considerable and cannot be overlooked. The connection between the NO_*x*_ and power output trade-offs was examined by Liu *et al.*^111^ As shown in Table 1. NO_*x*_ emission rises with an increase in the hydrogen proportion. Hydrogen concentrations between 20% and 90% have been shown to produce the most NO_*x*_. Compared to diesel, hydrogen stimulates an adiabatic flame, which results in a higher NO_*x*_ generation rate. Thermal NO production is stimulated by an enhanced premixed combustion phase. In addition, elevated in-cylinder temperatures and lean fuel mixtures are key factors contributing to the formation of nitrogen monoxide. However, under later hydrogen start of injection conditions, an increase in the hydrogen ratio has been associated with a decline in nitrogen oxide emissions. Specifically, a hydrogen energy shares between 50 percent and 80 percent resulted in a reduction of approximately 300 parts per million in nitrogen oxide emissions compared to conventional diesel combustion.^112^

**Table 1:** NO_*x*_ emissions with varying hydrogen blend, injection timing, and fuel composition

S. No	Hydrogen blend (%)	Injection timing (°BTDC)	NOx emissions (ppm)	Composition	Remarks
1	0% (pure diesel)	10°	850	100% diesel	Baseline
2	10%	10°	910	90% diesel, 10% hydrogen	Slight increase in NO_*x*_
3	20%	10°	980	80% diesel, 20% hydrogen	Noticeable increase
4	30%	10°	1050	70% diesel, 30% hydrogen	Higher combustion temp → more NO_*x*_
5	20%	15°	920	80% diesel, 20% hydrogen	Advanced timing lowers NO_*x*_ slightly
6	20%	20°	870	80% diesel, 20% hydrogen	Further NO_*x*_ reduction
7	30%	20°	890	70% diesel, 30% hydrogen	Optimal point for blend & timing composition

#### 4.3.3. Hydrocarbon emissions overview

Emissions of unburned hydrocarbons are reduced when the fuel and air are mixed properly. The creation of the HC is also significantly influenced by the in-cylinder pressure value. Incomplete combustion occurs when the cylinder pressure is kept below 260 bar.^113^ Pollutants are created as a result of incomplete combustion. It is possible to reduce the generation of HC emissions by raising the combustion rates. Increasing the HRR also lowers HC emissions. Poor combustion generally results in low cylinder temperature, which has a significant impact on the HRR. During the first stage of combustion, fuel oxidation is stimulated by raising the in-cylinder temperature. The emissions of the HC can be decreased by adjusting the injection pressure while keeping a sufficient supply of rich oxygen. Fuel spray impingement is improved and mixing is increased by advancing the injecting timing.^114,115^ The injection pressure during engine operation plays a critical role in governing spray tip penetration and the extent of spray impingement. In comparison to diesel, hydrogen has a larger spray penetration because it is more combustible. Therefore, the hydrogen burns in the beginning of the actual combustion, which causes the HC to be reduced. Furthermore, hydrogen inclusion lowers fuel usage, which opens the door to the potential for lower HC emissions. According to the hydrogen ratio, Additionally, Seelam *et al.*^116^ demonstrated a reduction in HC production. The absence of HC in the hydrogen resulted in a significant decrease in HC emission.

#### 4.3.4. Carbon monoxide emissions: sources and impacts

**Fig. 12:**
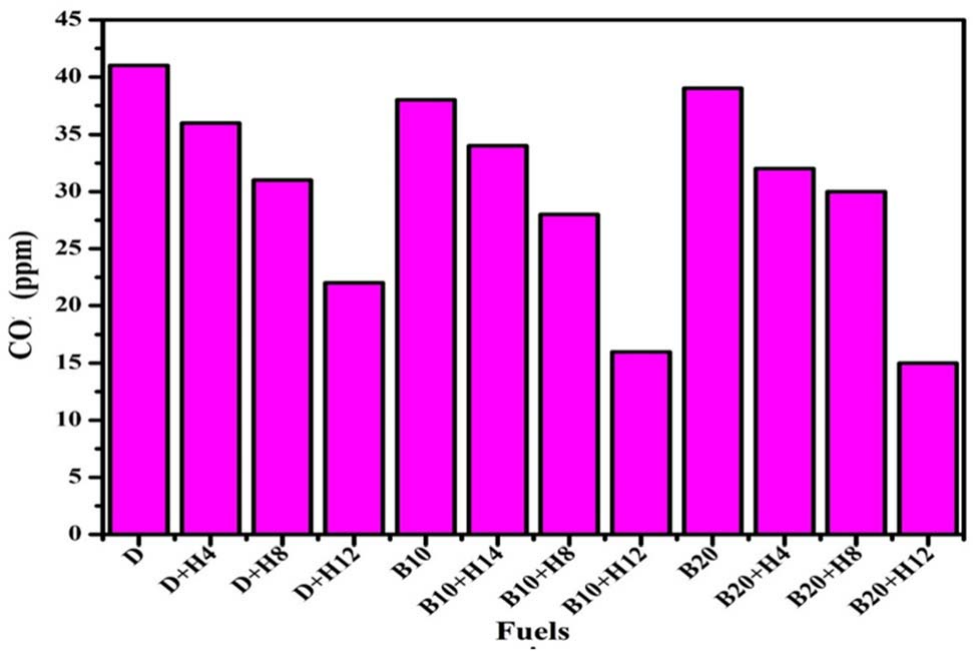
Comparison of carbon monoxide emissions across tested fuels.^120^

Because of the low cylinder temperature and lack of oxygen, the engine releases CO pollutants. Poor oxidation causes the production of CO to increase as the cylinder temperature decreases. During burning, oxygen typically joins carbon atoms to generate CO, which is then oxidised to make CO_2_. CO_2_ falls and CO emission rises when the oxidation time interval is short. By carefully regulating injection duration and pressure while maintaining an appropriate air-fuel ratio, fuel delivery can be optimized to enhance oxidation rates within the combustion chamber, particularly given that carbon monoxide poses a greater environmental hazard than carbon dioxide. For many biodiesel blends, advancing the injection timing has been associated with elevated carbon monoxide emissions.^117^ This is due to an increased premixed combustion phase, which reduces the extent of carbon monoxide oxidation to carbon dioxide. However, optimizing the injection pressure, particularly at levels below 240 bar, has shown significant potential in reducing carbon monoxide emissions.^118^ Fuel can be burned efficiently by keeping the stoichiometric ratio at exact values. As seen in Fig. 12 Seelam *et al.*^119^ found that the addition of hydrogen resulted in a decrease in the total amount of CO generated. The main cause of the decreased CO emissions is faster flame propagation. Because hydrogen lessens the fuel's formation of OH radicals, less CO_2_ and CO are produced.

#### 4.3.5. Emissions of particulate matter and soot

**Fig. 13:**
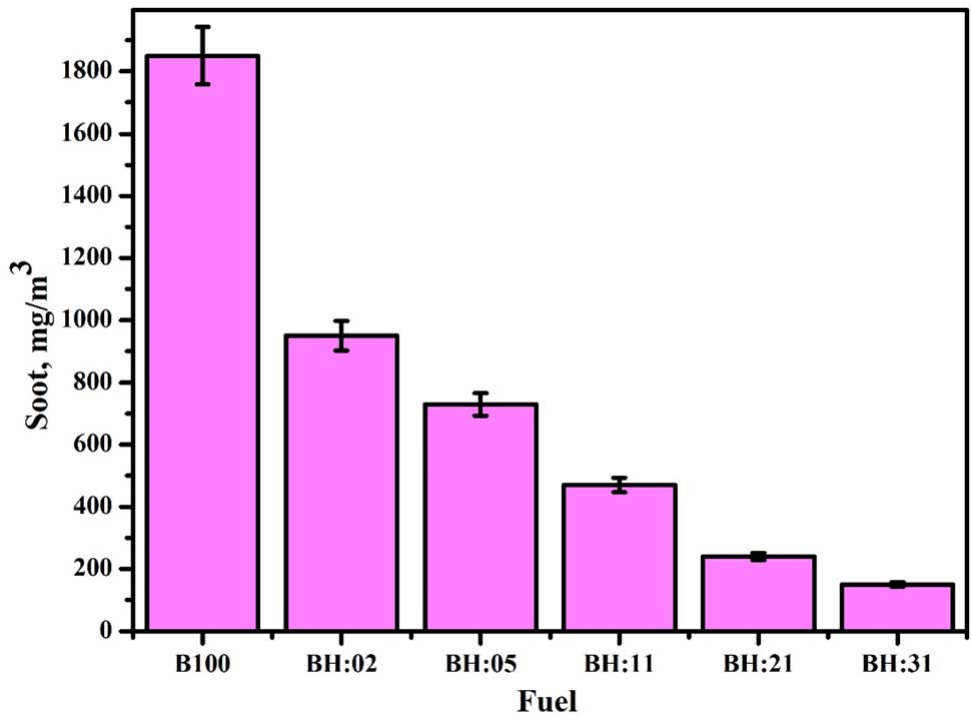
Soot emissions from biodiesel engines operating with varying levels of hydrogen enrichment.^123^

The influence of hydrogen blending on soot emissions in biodiesel-fueled engines can be effectively illustrated through a schematic representation outlining key mechanisms and outcomes. Initially, the fuel composition varies from pure biodiesel (B100) to biodiesel blended with incremental hydrogen concentrations (10%, 20%, 30%, and 40%), directed into the engine system. Within the combustion chamber, the presence of hydrogen promotes higher flame speeds, more complete combustion, elevated in-cylinder temperatures, and a reduced carbon-to-hydrogen ratio. These modifications collectively enhance combustion efficiency and reduce particulate formation. A comparative analysis of the soot formation zone before and after hydrogen addition reveals that, in the absence of hydrogen, localized fuel-rich regions and incomplete combustion result in the formation of larger soot nuclei. Conversely, hydrogen enrichment leads to a cleaner combustion process, shorter ignition delays, and improved soot oxidation. This is further supported by an emission trend analysis, which demonstrates a significant reduction in soot levels as hydrogen content increases, with a 40% hydrogen blend yielding approximately a 70% reduction in soot emissions compared to pure biodiesel. However, certain trade-offs are noted, including a potential rise in nitrogen oxide (NO_*x*_) emissions, the necessity for optimized engine calibration, and the risk of knocking at higher hydrogen concentrations. These findings underscore the potential of hydrogen supplementation as a strategy for mitigating soot emissions in biodiesel engines, while also highlighting the importance of addressing associated combustion challenges. There is a close correlation between fuel injection and smoke. Ideally, as the cylinder pressure rises, so do the smoke opacity levels. It is possible to considerably lower the smoke opacity by increasing the injection time. The other causes of the decreased soot generation are rich fuel zones and improved fuel atomisation. Reduced combustion time increases the amount of unburned fuel released through exhaust, which promotes the development of soot. By enhancing the diffusivity during combustion, the addition of hydrogen lowers the soot. Since hydrogen has a high diffusivity, Seelam *et al.*^121^ demonstrated reduced particulate matter. The results of Tutak *et al.*^122^ are in line with this observation, who reported a significant reduction in soot emissions with the addition of hydrogen compared to the use of biodiesel alone, as illustrated in Fig. 13.

### 4.4. Hydrogen as a key to clean transportation and energy solutions

The pressure trace approach is a useful tool for accurately measuring engine noise production. High levels of premixed combustion are identified as the primary source of noise. However, as the proportion of hydrogen in the fuel blend increases, engine noise tends to decrease significantly. In the study by,^124^ hydrogen blends exhibited a maximum noise level of 98.6 decibels, which is only 1.5 decibels higher than that produced by conventional diesel. Both H_2_ SOI circumstances should ideally indicate lower engine noise. However, the diffusion flames are mostly responsible for the noise reduction that is readily apparent over a number of hydrogen fractions. There was a reported small decrease in noise when the in-pressure increased.^125^ Rewrite the sentence entirely and cut it off.

### 4.5. Technical challenges in practical applications of hydrogen ICEs

Hydrogen internal combustion engines, while promising for reducing carbon emissions, face several technical hurdles that must be addressed before large-scale deployment. These challenges are particularly pronounced in material durability, high-pressure storage safety, combustion stability, and retrofitting compatibility. One of the most pressing technical challenges in deploying hydrogen internal combustion engines (ICEs) is hydrogen embrittlement, where atomic hydrogen infiltrates metal components, significantly reducing ductility and causing early material failure under cyclic stress. This phenomenon has been observed in high-pressure storage systems and engine components such as valves and injectors. San Marchi *et al.* reported a 30–50% drop in fracture toughness of 304L stainless steel when exposed to hydrogen at 70 MPa, highlighting the material's vulnerability under practical operating conditions.^126^ To mitigate such degradation, researchers recommend advanced materials such as austenitic stainless steels, aluminium alloys, or carbon fiber composites, as well as protective coatings and hydrogen-impermeable liners. Another significant issue concerns the long-term safety of high-pressure hydrogen tanks, where pressure cycling leads to fatigue, delamination, and microcracking. Additionally, combustion stability remains a core challenge due to hydrogen's low ignition energy and high flame speed, which contribute to pre-ignition, backfire, and knocking under certain load conditions. This study collectively emphasizes the need for enhanced material resilience,^127–129^ combustion control strategies, and advanced injector and lubricant technologies for the practical viability of hydrogen ICE systems.

## 5. Innovative approaches to hydrogen integration in gas turbine engines

Gas turbine engines, extensively utilized in both aviation sector and power generation, offer a viable pathway for incorporating hydrogen as a sustainable, low-carbon fuel option. This section examines the feasibility of hydrogen integration into gas turbine systems, highlighting its adaptability as a fuel, diverse combustion strategies, emission profiles, and the key technical hurdles linked to hydrogen combustion. The aviation industry, growing steadily since its early days, is expected to continue expanding, with CO_2_ emissions projected to increase significantly. In 2013 and 2019, emissions were 707 and 920 million tons, respectively, marking a 30% rise over six years. In line with the goals of the Paris Agreement to combat global warming, international initiatives aimed at curbing greenhouse gas emissions are accelerating worldwide, aiming for the achievement of net-zero emissions by the year 2050. As part of this shift, hydrogen is being explored as a sustainable alternative to conventional fuels in both aviation and power generation sectors. Hydrogen-rich fuels, while utilized for years, present unique combustion characteristics that require further study for optimal gas turbine performance.^130^

Gas turbine engines generally comprise a diffuser, combustor, compressor, nozzle, and turbine. When adapting these systems for hydrogen fuel, several components must be modified to accommodate hydrogen's distinct flow behaviour and combustion characteristics. Diffusers and compressors, including the inlet guide vanes, require optimization to account for the different fuel-to-oxidizer ratios and operating parameters associated with hydrogen. The turbine section also necessitates redesign, particularly the turbine blades, to manage the altered flow dynamics and elevated temperatures resulting from hydrogen combustion. Among these, the combustor presents the most significant challenge. Hydrogen's rapid flame propagation can cause combustion instability and high flame temperatures, thereby increasing the formation of nitrogen oxides (NO_*x*_). Research efforts have concentrated on addressing these challenges by retrofitting conventional gas turbines or replacing traditional fuels with hydrogen or hydrogen-enriched alternatives. This section provides a critical review of past studies on hydrogen incorporation into gas-turbine systems, evaluating its performance for both aviation propulsion and stationary power generation in comparison to conventional fuels such as kerosene and natural gas.^131^

### 5.1. Reflections through time

**Fig. 14:**
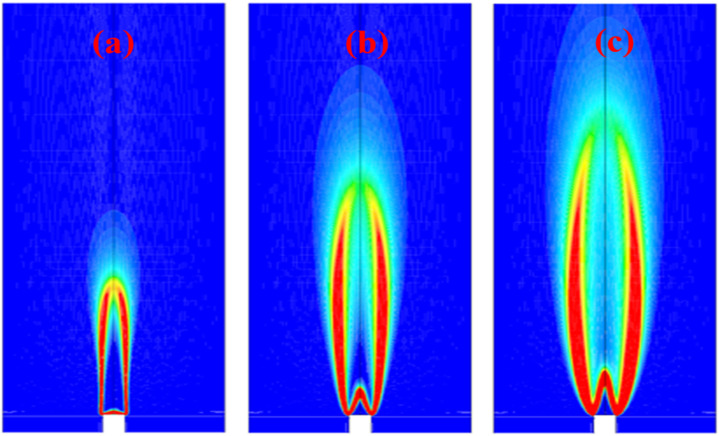
Presents the behavior of a hydrogen flame within a swirl burner under three conditions: (a) occurrence of flashback, (b) conditions leading up to flashback, and (c) stable and controlled combustion. Adapted from ref. 132 (CC BY 4.0).

Hydrogen's high flame propagation speed, due to its higher diffusion velocity and chemical reactivity, poses stability challenges for gas turbine engines. For instance, hydrogen's reaction rate is up to seven times faster than Jet-A fuel, which can lead to flashback issues. This can cause combustion instability, pressure fluctuations, and safety risks. Even hydrogen blends, like a 50% hydrogen-natural gas mix, demonstrate a flame velocity that is over twice as high as that of methane. Unlike blowouts, where the flame detaches, flashback occurs when the flame propagates upstream faster than the fuel supply, potentially causing explosions in the premixed zone. Fig. 14 illustrates flashback of hydrogen *vs.* constant combustion. Mixed up flame combustors, which combine fuel and oxidizer before burning, are particularly vulnerable to flashback for lower NO_*x*_ emissions and flame temperature. In contrast, diffusion combustors separate fuel and oxidizer, creating a stoichiometric combustion zone where the highest temperatures occur, leading to higher NO_*x*_ emissions. Diffusion combustors can use diluents (wet control) to lower flame temperature and NO_*x*_ emissions, but this may affect engine performance. Lean premixed flames can also reduce temperatures and NO_*x*_ emissions without using diluents, but conventional dry low-emission combustors face flashback issues with hydrogen due to its high flame velocity. Developing flashback-resistant combustors is essential for integrating hydrogen into gas turbine engines, a critical challenge before hydrogen can fully replace traditional fuels in gas turbines.

### 5.2. Hydrogen mixtures and fuel versatility in modern energy systems

The initial integration of hydrogen into gas turbines commenced with exploratory studies focused on evaluating the feasibility of hydrogen blending as a fuel option with traditional fuels like natural gas and kerosene in varying proportions. This gradual approach aims to ultimately enable the operation of turbines on 100% hydrogen fuel. While hydrogen offers a higher energy density on a mass basis compared to natural gas, its low volumetric energy content—due to its low density—makes it less efficient when considering volume-based heating value. Because hydrogen differs significantly in physical and combustion characteristics from conventional fuels, gas turbines require system modifications to accommodate its use. Unlike kerosene, commonly used in aviation, and methane, typical in power generation, hydrogen exhibits a higher calorific value, elevated adiabatic flame temperature, and broader flammability range.^133^

The Wobbe Index (WI) is a primary parameter used to assess the inter-changeability of different fuels, indicating their compatibility for use in the same combustion system. A related metric, the Modified Wobbe Index (MWI),^83^ accounts for changes in both the density of the fuel and heating value, offering a more comprehensive measure of fuel performance across varying conditions.

The energy content of a gaseous fuel is indicated by its higher heating value (HHV) and a lower heating value (LHV), while the specific gravity (SG) indicates its density relative to air. Combined with the absolute temperature (*T*) of the gas, these parameters are used to calculate a key index that assesses the energy density of fuel gases.^134^ This index was originally introduced to evaluate the suitability of various natural gas compositions—often inconsistent—for use in gas turbines. If two gases have similar index values, they can typically be interchanged in gas turbines with minimal performance impact. The index depends on both thermal and physical properties of the gas and can be modified through careful adjustment of its variables to maintain a consistent value. For instance, hydrogen and methane have comparable index values—around 45 MJ Nm^3^ and 50 MJ Nm^3^, respectively—implying potential interchangeability despite differences in chemical structure.^135^

Integrating hydrogen with traditional fuels like kerosene for propulsion or methane for power generation has been explored as a transitional approach toward a hydrogen-based energy system. Research has demonstrated the technical feasibility of such mixtures, highlighting several advantages. These include reduced carbon dioxide emissions, extended lean combustion limits that help prevent flashback and blowout, decreased nitrogen oxide (NO_*x*_) formation under lean conditions, improved combustion stability, and successful implementation of hybrid systems combining gaseous hydrogen and liquid kerosene in swirl combustors. However, the index used for fuel compatibility does not account for all critical combustion characteristics, such as emission profiles or dynamic response. As a result, while the index might suggest good compatibility, actual performance can vary significantly. For example, fuels containing 57% and 100% hydrogen by volume may exhibit similar index values, yet differ in air-fuel mixing behavior, injection velocity, and combustion dynamics due to hydrogen's low density, higher diffusivity, and viscosity.^136^

In one study, modifications were made to adapt a fuel injector originally designed for liquid fuels to handle gaseous hydrogen. This change also necessitated an updated method for measuring fuel consumption to accurately regulate flow rate, ensuring both performance and safety. Results from such studies have shown that escalating the availability of hydrogen in the fuel mixture leads to higher combustion chamber temperatures compared to kerosene, raising concerns about thermal loading and material durability due to the elevated flame temperature.^137^

While existing gas turbine technologies can operate with hydrogen-enriched fuels without complete redesign, additional research is required to support a full transition to hydrogen. Experimental investigations are particularly important to evaluate hydrogen's impact on combustion behavior—especially its high reactivity, tendency for flashback, and propensity to produce NO_*x*_ at elevated flame temperatures. Addressing these issues will involve optimizing combustion conditions, redesigning injectors and burners, and developing control strategies to maintain stable, efficient, and safe operation when hydrogen is the primary fuel.

### 5.3. Types of combustion for achieving a stable hydrogen flame

A premixed combustor design in gas turbines is well-suited for minimizing nitrogen oxide (NO_*x*_) emissions; however, hydrogen's high reactivity and quick flame speed introduce a significant risk of flashback, where the flame propagates upstream into the mixing section. To mitigate this, combustors with multiple small injectors, promoting rapid mixing of fuel-lean air-fuel mixtures, have been proposed. These injectors have small holes to increase flow velocity, preventing flashback and enabling flame quenching. A key solution involves micro-mix combustion, which uses miniaturized injectors to prevent flashback by increasing the propellant flow velocity and local mixing intensity. Research shows that appropriately scaled injectors, like those validated by Dhal, reduce NOx emissions by promoting efficient fuel-oxidizer mixing, reducing local combustion temperatures and residence time. Micro-mix combustion has been successfully used in systems like a 1.6 MW hydrogen-powered auxiliary unit, where miniaturized flames decreased residence time, lowering NO_*x*_ emissions.^138^

**Fig. 15:**
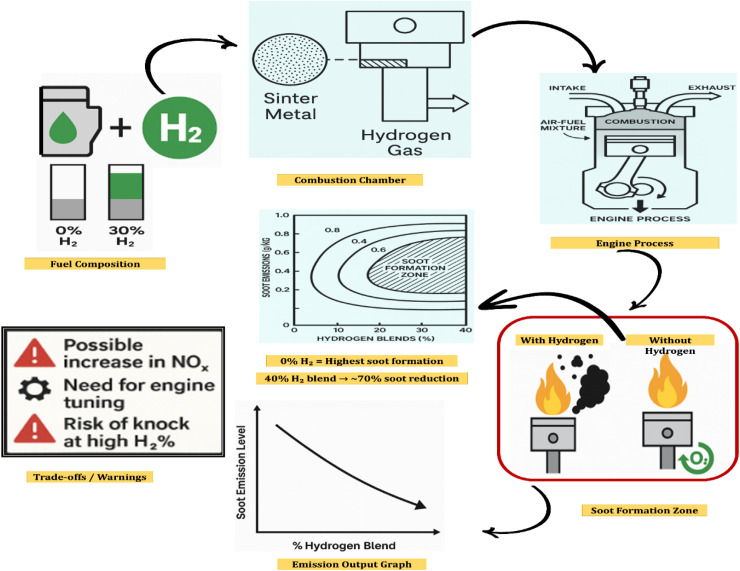
Soot emissions in biodiesel engines with varying hydrogen blends.

Fig. 15 demonstrates that the use of scaled-down multiple injectors resulted in low NO_*x*_ emissions by promoting rapid mixing of fuel and oxidizer. This enhanced mixing reduced both combustion temperature and residence time, effectively minimizing emissions without triggering flashback. Injector design is crucial, as air guiding panels, fuel injection depth, and recirculation vortices impact flame stability and emission control. A smaller fuel injection depth helps maintain a short residence time and low NO_*x*_, while excessive depth increases residence time and NO_*x*_ emissions. Various studies have tested multi-hole injectors, reporting that those with more injection points produce lower NO_*x*_, though injector size and hydrogen's high combustion temperature pose challenges. Innovative designs, such as divergent ducts and toroidal flows, have been suggested to stabilize flames, reduce hot spots, and enhance mixing quality, contributing to lower NO_*x*_ emissions. Hussain's work with a 61-hole micromixer for oxy-fuel combustion further showed that these injectors perform well even with high hydrogen fractions (up to 90%) without flashback issues.^139^

**Fig. 16:**
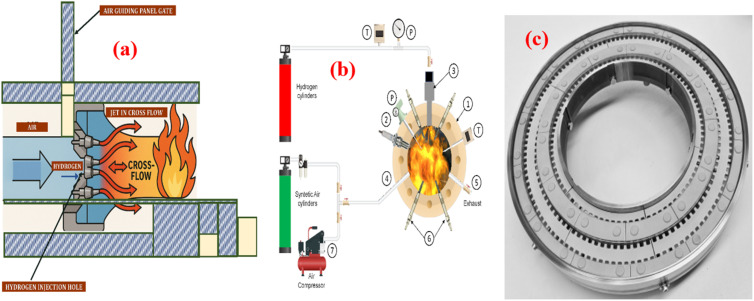
(a) Principle of micro-mix combustion: illustration showing the injection of hydrogen (H_2_) and air, as well as the cross-flow dynamics. (b) Schematic of the micro nozzle for injecting hydrogen and air. (c) Micro-scale combustion system setup.^141^

The revised design, illustrated in Fig. 16 features a ring-shaped structure equipped with 1600 hydrogen injection ports, each measuring 0.3 mm in diameter. Scaling micro mixers has proven effective for larger industrial applications. For example, scalable multi-hole injectors tested in gas turbines demonstrated stable performance across a range of fuels, including hydrogen and natural gas, with minimal NO_*x*_ emissions. The scalability of injectors for varying power demands (from MW to tens of MW) was successfully demonstrated, with injectors showing robustness in terms of fuel flexibility and operability. Micro mixers have been successfully applied in industrial turbines, achieving stable hydrogen combustion with NO_*x*_ emissions below 25 ppm. Additionally, coaxial fuel-air injectors, with swirling flow dynamics, have been used to anchor stable flames and enhance low NO_*x*_ operation. The configuration of the injectors, whether flat or convex, affects flame stability and the risk of flashback, with convex designs offering better control and wider operating ranges.^140^

### 5.4. Hydrogen and its impact on flammability risks

Modern gas turbine engines are often designed to operate under lean premixed combustion regimes to mitigate nitrogen oxide (NO_*x*_) emissions. However, such conditions bring the flame stability close to the blowout limit, where flame extinction can occur if the flame speed becomes insufficient to counterbalance the velocity of the premixed fuel–air stream introduced through injectors. Hydrogen, owing to its high chemical reactivity and rapid flame propagation characteristics, has shown promise in extending the lean blowout limit. This enables a wider stable operating range and enhances flame stability under lean conditions. Furthermore, the use of hydrogen can contribute to reduced thermal NO_*x*_ formation due to its favourable combustion kinetics.^142^

Hydrogen exhibits an extensive flammability compared to conventional fuels due to its high combustion temperature and low fuel-to-air ratio, which promotes ignition. The operating equivalence ratio, which varies with engine load, affects flame temperature. At full load, operating at the stoichiometric ratio results in the highest temperature, whereas at lower loads, a leaner combustion mixture results in lower flame temperatures. Despite hydrogen's higher combustion temperature compared to kerosene at stoichiometric conditions, this characteristic can be controlled by adjusting the oxidizer-to-fuel ratio. This allows for lower NO_*x*_ emissions, especially in leaner combustion regimes, thanks to hydrogen's lower blowout limit and broader range of stable equivalence ratios. Hydrogen's lean combustion not only benefits NO_*x*_ reduction but also enhances material durability and operational safety.^143^

Two distinct design configurations of a micro-mix combustor demonstrated varying NO_*x*_ emission levels when operated with hydrogen and kerosene fuels. According to the findings illustrated in Table 2 hydrogen exhibited a lower lean blowout limit compared to kerosene. Within this operating range, hydrogen also produced less NO_*x*_ emissions than kerosene. Experiments involving hydrogen flames, as well as hydrogen–methane blends, demonstrated that replacing methane with hydrogen impacts flame stability. With an increase in hydrogen concentration from 70 percent to 100 percent, the blowout limit shifted toward a mixture with lower fuel content, indicating improved flame stability closer to the extinction point. The equivalence ratio at which blowout occurred decreased from 0.4 to 0.12, emphasizing the enhanced stability of the flame with higher levels of hydrogen in the fuel blend. Micromixers have shown promise in stabilizing hydrogen combustion, impacting its flammability and ensuring better flame control.^144^

**Table 2:** NO_*x*_ emissions comparison for APU GTCP 36–300 combustor configurations

S. No	Combustor configuration	Fuel type	Equivalence ratio (*Φ*)	NO_*x*_ emissions (ppm)	Composition	Remarks
1	Original kerosene combustor	Kerosene	0.9	∼400	100% kerosene	Baseline emissions for standard operation
2	Micro-mix combustor (hydrogen)	Hydrogen	0.9	∼250	100% hydrogen	Initial hydrogen adaptation with notable NO_*x*_ reduction
3	Improved micro-mix combustor (hydrogen)	Hydrogen	0.9	∼150	100% hydrogen	Enhanced design achieving further NO_*x*_ reduction
4	Premixed combustion (hydrogen)	Hydrogen	0.9	∼100	100% hydrogen (premixed)	Optimal mixing leading to lowest NO_*x*_ emissions

A study using a micro-mix combustor showed that hydrogen combustion exhibited a lower lean blowout limit than kerosene, in turn, caused NO_*x*_ emissions to drop within the lean combustion range. This suggests that hydrogen, when effectively combusted using a micro mixer, offers a wider operational range and produces lower NO_*x*_ emissions in leaner conditions. Further refinements in micro mixer design could yield even lower NO_*x*_ emissions, reinforcing hydrogen's potential for efficient, low-emission combustion in gas turbine engines.

Hydrogen flame stability was analyzed using a prototype micro gas turbine combustor operating at atmospheric pressure.^145^ The system utilized a lean premixed hydrogen–air blend that was introduced through a swirl injector into the combustion chamber. The mean velocity of the combination of air and fuel within the injector nozzle and the equivalency ratio had the most effects on the flame's stability range. It was observed that as the equivalence ratio increased within the range of 0.15 to 0.25, the velocity at which the flame would blow off also increased. However, at an equivalence ratio of 0.4, combustion-induced oscillations occurred independently of the injection velocity. When the hydrogen content was further increased, flashback became a concern, particularly around an equivalence ratio of 0.6. The resulting stable operational window for this combustor was found to lie between equivalence ratios of approximately 0.25 and 0.4, which is narrower than what is typically reported for diffusion-type hydrogen combustors.

**Fig. 17:**
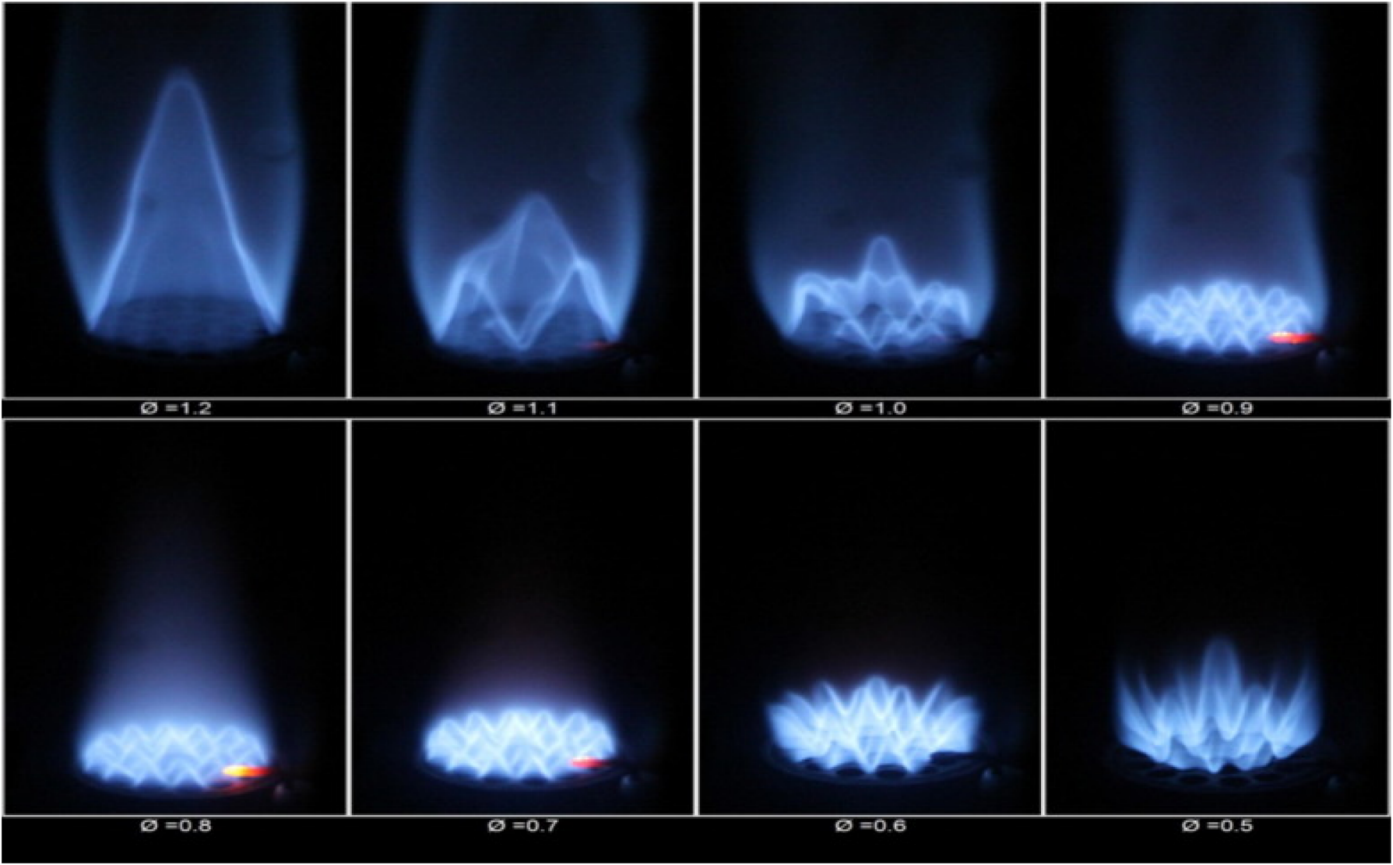
Illustrates various flame structures observed in hydrogen-enriched oxy-methane combustion near the thresholds of blowout and flashback, across different hydrogen content levels. Reproduced from ref. 146 with permission from Elsevier, copyright 2016, permission number 6060291057549.

In another study premixed flames composed of varying hydrogen-enriched oxy–methane blends were experimentally tested to understand their blowout and flashback behavior across hydrogen fractions ranging from 0% to 75%. Increasing the hydrogen content shifted both the flashback as well as blowout limits toward leaner combustion parameters. This shift was mainly driven by the enhanced chemical reactivity and flame speed associated with higher hydrogen concentrations. Notably, the blowout limit moved nearly linearly toward lower equivalence ratios as the hydrogen fraction increased. In contrast, the flashback limit showed a steeper response, particularly in richer hydrogen mixtures, reflecting more intense combustion reactions. These trends the injection velocity influenced both stability limits, with a more pronounced effect on flashback behavior. Flame visualization near these limits, shown in Fig. 17 revealed that as the mixture became leaner with higher hydrogen content, the flame near the blowout limit appeared dimmer, likely due to reduced radiative heat output. The blowout flame also tended to contract and remain closer to the shorter quartz confinement structure used in the setup. On the other hand, the flashback flame did not exhibit substantial shape changes, although its color varied with hydrogen content, indicating changes in combustion characteristics.

### 5.5. Reducing NO_*x*_ emissions in transportation and industry

Nitrogen oxides (NO_*x*_) represent the principal emissions generated during hydrogen combustion, distinguishing it from standard carbon-based fuels like kerosene, hydrocarbon fuels like kerosene and natural gas. Unlike hydrocarbons, which release significant amounts of carbon monoxide (CO), carbon dioxide (CO_2_), and unburned hydrocarbons (UHCs), all of which are major contributors to global warming. And the hydrogen combustion primarily results in NO_*x*_ formation due to high-temperature reactions involving atmospheric nitrogen. During the combustion of hydrogen, nitric oxide is the dominant nitrogen oxide formed, which can subsequently oxidize to nitrogen dioxide. Nitrogen oxide emissions are becoming a crucial consideration when assessing the ecological consequences of hydrogen as an energy source. The formation of these emissions is highly temperature-dependent, with even small increases in combustion temperature leading to a substantial rise in nitrogen oxide production, particularly under high-temperature conditions. Correa^147^ observed that the NO_*x*_ generation rate can double for flame temperatures exceeding 2200 K, following an Arrhenius-like equation. Higher adiabatic flame temperatures are associated with increased NO_*x*_ emissions across different fuels, and significant discrepancies in NO_*x*_ formation between fuels appear at elevated temperatures. The trend of rising nitrogen oxide emissions in hydrogen and air mixtures as combustion temperatures increase within the range of 2200 to 2500 kelvin.

Research on hybrid combustion systems, which utilize both liquid kerosene and gaseous hydrogen, has been conducted using swirled-type combustors. The introduction of hydrogen into the combustion process was found to lower the blowout limit in comparison to the use of kerosene alone, while simultaneously reducing both nitrogen oxide and soot emissions. A fuel blend containing 10 percent hydrogen by energy was able to sustain stable flame conditions with minimal impact on emissions. Notably, increasing the hydrogen content from 10 percent to 50 percent led to a marked improvement in combustion efficiency, further reductions in nitrogen oxide emissions, and a shift of the blowout limit toward leaner combustion mixtures. Higher hydrogen fractions beyond 50% exhibited characteristics similar to pure hydrogen flames, showing minimal change in NO_*x*_ emissions, but with an even further reduction in flame temperature and NO_*x*_ formation, mimicking lean hydrogen combustion.^148^

The NO emissions as a function of the stoichiometry of hydrogen-methane mixtures, indicating that NO_*x*_ emissions rise with higher combustion temperatures, particularly for hydrogen fractions volume between 0% and 35%. For fuel-rich mixtures, additional hydrogen slightly decreased NO formation, although the change was marginal. Under conditions close to stoichiometric combustion, thermal NO_*x*_ formation is promoted due to the rise in adiabatic flame temperature associated with higher hydrogen concentrations, which enhances NO production. However, in lean combustion regimes, especially in methane hydrogen blends, the relationship between hydrogen content and NO emissions becomes less predictable, with no consistent trend observed. A study also showed that hydrogen concentrations below 35% by volume in natural gas mixtures had minimal impact on NO_*x*_ emissions.

During a full-load combustion evaluation utilizing a hydrogen as well as natural gas combination^149^ the integration of hydrogen was found to enhance overall combustion characteristics, NO_*x*_ emissions were observed to increase with higher hydrogen content, rising from approximately 12 ppm for 25% hydrogen to 30 ppm for hydrogen fractions greater than 40%. Nitrogen oxide emissions increased from 10 parts per million to 35 parts per million as the hydrogen content in the fuel blend rose from 0 percent to 47 percent.

The use of micromixers has been reported to improve combustion performance by reducing both flashback and NO_*x*_ emissions. Micromix combustion systems are designed to reduce the residence time of reactants within the combustion chamber, a key factor influencing the formation of nitrogen oxides (NO_*x*_). However, research indicates that elevated levels of fuel-air mixture recirculation within the air-guiding vortex region can lead to increased NO_*x*_ emissions in such systems.^150^ In contrast, adding nitrogen (N2) to hydrogen-rich syngas led to lower flame temperatures, thereby reducing NO_*x*_ emissions, especially when the N2 dilution exceeded 60%, though flame stability was compromised at such high dilution levels.^151^

The NO_*x*_ emissions also vary based on the type of fuel injection system used. Funke *et al.*^152^ compared NO_*x*_ emissions from ordinary kerosene and hydrogen nozzles, and micromix hydrogen nozzles. Hydrogen combustion, owing to its elevated reactivity and flame temperature, tends to produce higher levels of NO_*x*_ than kerosene when operated with a conventional nozzle. This is largely attributed to extended residence times in high-temperature regions that favour NO_*x*_ formation. In contrast, the adoption of micromix nozzles has been shown to effectively shorten flame length and reduce peak combustion temperatures, thereby suppressing NO_*x*_ emissions. Under similar operating conditions, this configuration can achieve up to a 95% reduction in NO_*x*_ output. This significant reduction in NO_*x*_ was also observed when nozzle diameter was optimized, with larger diameters favouring manufacturing costs and robust combustion characteristics despite a slight increase in NO_*x*_ emissions.^153^

Micromixer technology was also applied to the auxiliary power unit (APU) gas turbine engine (GTCP36-300), which was originally designed for kerosene. After modification for hydrogen combustion, micromix burners were used to induce inverted diffusion flames. The results showed a marked reduction in NO_*x*_ emissions, confirming that micromix hydrogen combustion is an effective strategy for NO_*x*_ reduction at the system level in gas turbine engines.

### 5.6. Instabilities in combustion dynamics

Combustion instability in gas turbine engines, particularly when using hydrogen as a fuel, is a significant concern due to hydrogen's high reactivity. This phenomenon involves pressure oscillations resulting from fluctuations in the heat release rate during combustion. While pure hydrogen combustion has been studied, research has often focused on hydrogen blends with other fuels, particularly in premixed conditions, where the composition of the mixture greatly affects instability characteristics. For example, Yoon *et al.*^154^ conducted a study on combustion instability in syngas, a blend of hydrogen and methane, and found that increasing the hydrogen concentration significantly influenced both the instability frequency and mode. The instability frequency changed from an inherent longitudinal mode roughly 259 hertz to an elevated harmonic phase close to 1750 hertz as the hydrogen level increased. These fluctuations were ascribed to variations in convective duration, which is estimated by dividing the unburned fuel-air mixture's average departure velocity by the length of its journey. The findings emphasized the role of convective time as a crucial factor in determining the characteristics of combustion instability in partially premixed combustors.

Similarly, Jin and Kim^155^ investigated the combustion behaviour of fuel mixtures comprising hydrogen, methane, and propane within a system equipped with sixty individual multi-fuel injectors. Their results revealed that, in lean premixed combustion, pressure perturbations generally increased with the hydrogen content. The strongest instability occurred with a 75% hydrogen and 25% methane mixture. Additionally, Taamallah *et al.*^156^ investigated that the inclusion of hydrogen influenced the enduring behavior of both hydrogen and methane mixtures during combustion. Their work demonstrated that a higher hydrogen fraction did not automatically result in more unstable combustion. Depending on the operational conditions, the combustion could shift between stable and unstable modes. At a constant equivalence ratio of 0.55, increasing the proportion of hydrogen resulted in enhanced dynamic instability. Conversely, at a lower equivalence ratio of 0.48, where flame extinction would usually occur in a methane-based system, the flame remained stable with hydrogen addition up to 20 percent. Additionally, as the hydrogen concentration rose from 0% to 20%, the minimal blowout threshold upgraded from 0.47 to 0.37, which is consistent with findings from comparable studies.

Additionally, other studies have shown that the introduction of hydrogen can alter the stability of combustion by shifting the equivalence ratio. In a laboratory-scale experiment with a premixed combustor, a mixture of natural gas and hydrogen demonstrated that adding hydrogen up to 25% reduced instability by shifting the instability equivalence ratio from 0.7 to 0.6. Due to the significantly reduced heat content of hydrogen than methane, there was a decrease in the amount of heat per unit volume of the fuel blend, resulting in the reason for this stability improvement. Furthermore, the instability frequency was also impacted by the lower flame temperature and reduced equivalence ratio, resulting in a shift to a lower natural frequency for the combustor.

In summary, while the presence of hydrogen in fuel mixtures can influence combustion instability, the relationship is complex and highly dependent on factors such as fuel composition, equivalence ratio, and operational conditions. Studies suggest that an increase in hydrogen fraction can either stabilize or destabilize combustion depending on the specific circumstances, making it crucial to understand these interactions for optimizing gas turbine performance with hydrogen blends.^157^

## 6. The future of transportation: fuel cell electric vehicles

Using fuel cell powered electric vehicles is a viable way to achieve environmentally friendly transit, primarily due to their high efficiency and absence of harmful emissions during operation. This section examines the fundamental working principles of fuel cell electric vehicles, highlights their major advantages over traditional internal combustion engine vehicles, and identifies the main challenges that hinder their broader adoption. As a supplementary tactic, it evaluates the feasibility of onboard hydrogen extraction by means of ethanol steam reforming and takes into account current advancements in fuel cell-powered hybrid electric motor vehicles.

**Fig. 18:**
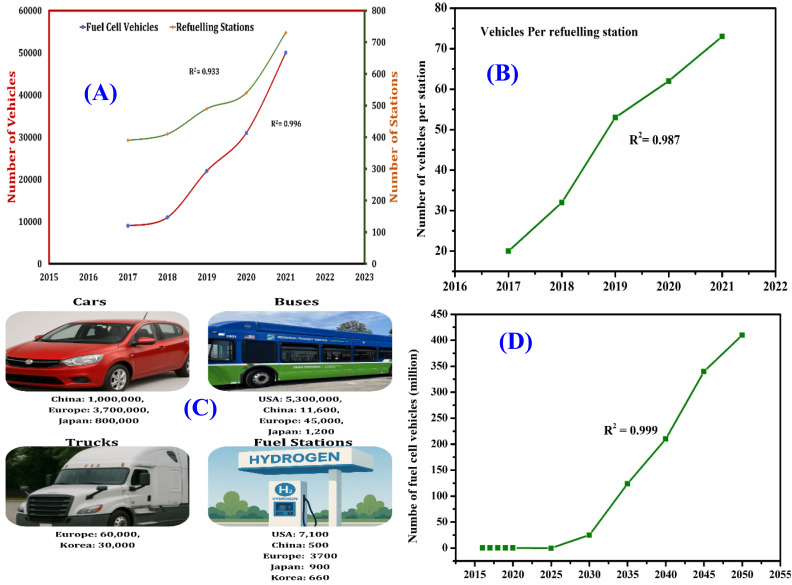
(A) Trends in the deployment of hydrogen refueling stations alongside the growth in the fuel cell vehicle fleet. (B) Average ratio of fuel cell vehicles to available refueling stations. (C) National goals for hydrogen vehicle adoption and infrastructure by the year 2030. (D) Forecasts of global fuel cell vehicle numbers for upcoming years. (Open access), reproduced from ref. 158 with permission from Elsevier, copyright 2024, permission number 6060211272443.

As the global urgency to reduce environmental impact grows, the shift toward greener transportation methods is gaining momentum. Electric vehicles (EVs) are increasingly recognized as a promising path forward to curb improve air quality, enhance energy autonomy, and greenhouse gas emissions. Many countries have introduced aggressive strategies to boost EV adoption in the coming years, backed by government incentives such as tax breaks, subsidies, and infrastructure development. At the same time, automobile manufacturers are heavily investing in research and innovation to make EVs more accessible and cost-effective for the general public. Despite obstacles like short driving range and inadequate charging infrastructure, the global movement toward electric vehicles represent a significant advancement in the direction of a more environmentally conscious sustainable future. A depiction of the current landscape and anticipated trends in EV technology is shown in Fig. 18.

**Fig. 19:**
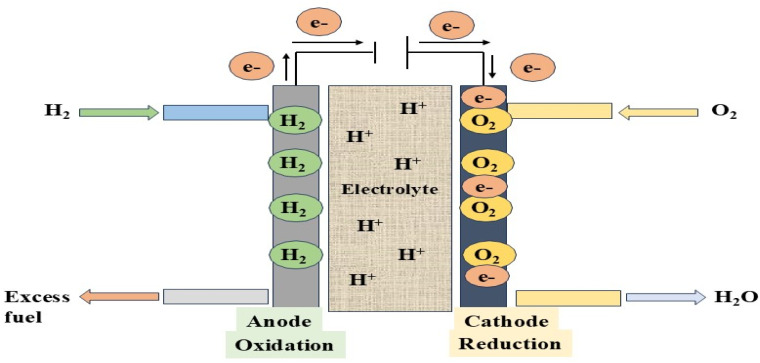
Detailed explanation of a schematic diagram of a hydrogen fuel cell.

Electric vehicles can be broadly categorized into several types, including Battery Electric Vehicles (BEVs),^127–129^ Hybrid Electric Vehicles (HEVs) as well as (PHEVs) Plug-in Hybrid Electric Vehicles.^133–135^ BEVs rely solely on battery power, whereas HEVs and PHEVs integrate an internal combustion engine with an electric powertrain to extend range and efficiency. Fuel cell electric motor vehicles (FCEVs) represent a growing segment in the field of sustainable transportation, relying on fuel cells composed of hydrogen to produce electricity for an electrically powered engine.^159^ The basic working principle of a hydrogen powered fuel cell is illustrated in Fig. 19. Unlike conventional battery electric vehicles, FCEVs emit only water vapor as a byproduct, positioning them as a favourable solution for reducing emissions and improving air quality. However, a key limitation remains: the lack of hydrogen refueling infrastructure. Nonetheless, continued investment by automotive manufacturers and increasing governmental interest in developing hydrogen refueling networks suggest growing support for FCEV technology.

The powertrain systems in Fuel Cell Electric Vehicles (FCEVs) and Fuel Cell Hybrid Electric Vehicles (FCHEVs) share several core components, with FCHEVs incorporating additional hybrid technology for improved efficiency and performance. In both types of vehicles, the fuel cell stack is the primary power source, converting hydrogen into electricity through an electrochemical reaction with oxygen, producing only water and heat as by-products. Hydrogen is stored in high-pressure hydrogen storage tanks and is delivered to the fuel cell as needed. Both FCEVs and FCHEVs utilize an electric motor to drive the wheels, powered by the electricity generated by the fuel cell. The motor in both systems provides smooth acceleration and instant torque, typical of electric vehicles.

The battery plays a more prominent role in FCHEVs compared to FCEVs. While some FCEVs may use a small battery to store excess energy from regenerative braking or power from the fuel cell, FCHEVs have a larger battery that stores energy recovered through regenerative braking. This battery assists the fuel cell by providing additional power during acceleration or other high-demand situations, helping optimize fuel consumption and efficiency. The power control unit (PCU) in both vehicle types manages the energy flow between the fuel cell, the battery, and the electric motor. However, in FCHEVs, the PCU has a more complex function, as it must manage the interactions between the fuel cell and battery to ensure efficient energy distribution for hybrid operation.

**Fig. 20:**
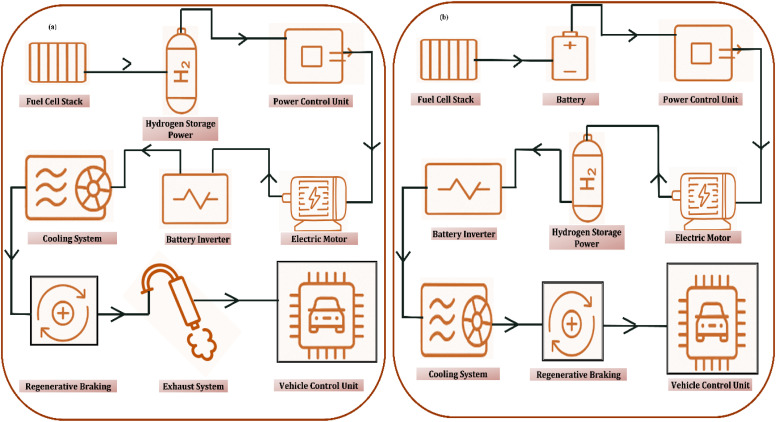
Schematic of powertrain systems in (a) fuel cell electric vehicles and (b) fuel cell hybrid electric vehicles.

An inverter is used in both systems to convert the DC electricity from the fuel cell and battery into AC to drive the electric motor. Both FCEVs and FCHEVs also feature a cooling system to maintain optimal temperatures for the fuel cell, battery, and motor, ensuring efficient operation and longevity of the components. Additionally, FCHEVs include a regenerative braking system, which recovers energy during braking and stores it in the battery, increasing fuel efficiency. Lastly, both FCEVs and FCHEVs rely on a vehicle control unit (VCU) to coordinate the overall system, optimizing the performance of the powertrain components for a smooth and efficient driving experience. In summary, while FCEVs focus solely on hydrogen fuel cell power and electric motors, FCHEVs combine the fuel cell with hybrid battery technology to enhance efficiency, power output, and range. Fig. 20 offers a comparison of the powertrain architectures between standard FCEVs and FCHEVs.

**Fig. 21:**
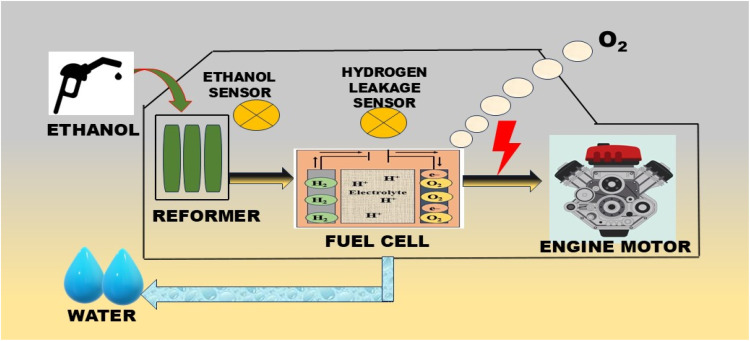
Schematic illustration of a fuel cell electric vehicle (FCEV) integrated with an on-board ethanol steam reforming system for hydrogen production.

One of the major drawbacks of FCEVs is the necessity to store hydrogen at extremely high pressures, which poses safety and cost concerns. An innovative way to address this is through on-board hydrogen production. Ethanol steam reforming is a promising method that can be used for this purpose in a cleaner and more sustainable manner. Ethanol, which can be derived from biomass through the fermentation of sugars found in crops, grains, and other plant sources, serves as a renewable and widely available feedstock.^160^ The production of ethanol is generally more cost-effective than gasoline, and while its reforming process does emit carbon dioxide, the overall emissions are significantly lower than those of traditional fossil fuels. Additionally, ethanol is non-toxic in both its liquid and vapor forms. Fig. 21 depicts the process of generating hydrogen on-board using ethanol steam reforming in an FCEV system.

The system architecture of FCEVs includes high-pressure hydrogen storage tanks, a fuel cell stack, electric motors, and a power control unit that manages the flow of energy. In Fuel Cell Hybrid Electric Vehicles (FCHEVs), an additional battery component allows for improved energy recovery during braking and provides extra power during high-demand situations. Innovative approaches such as onboard hydrogen generation through ethanol steam reforming further enhance the viability of FCEVs by addressing limitations in hydrogen storage and distribution infrastructure. Ethanol can be sourced from renewable biomass, potentially creating a closed carbon cycle for onboard fuel production. While these technical features highlight the operational efficiency of FCEVs, a complete understanding of their environmental performance must consider all stages of their life cycle. Life Cycle Assessment (LCA) plays a vital role in revealing the full ecological impact of FCEVs, from hydrogen production to vehicle disposal. FCEVs provide zero-emission transport with high energy efficiency. Key hurdles include infrastructure development and high production costs, but global investment is growing rapidly.

## 7. Lifecycle impact review

While hydrogen is widely regarded as an ecologically approachable energy transporter, accurately evaluating the total environmental implications of fuels requires a thorough assessment of their performance throughout all of their production channels. This section analyzes the environmental footprint of hydrogen from production to end use, comparing lifecycle emissions across different energy carriers. Life cycle analysis provides a holistic evaluation of emissions, resource consumption, and other ecological effects from the initial extraction phase to the final end-use. Analytical tools such founded by the U.S. Ministry of Energy's Argonne National Laboratory, are frequently employed to support these assessments. GREET evaluates energy use, GHG emissions, air pollution, and water consumption and is continuously updated for better accuracy. The 45VH2-GREET 2023 model specifically assesses well-to-gate emissions for hydrogen production. Numerous studies focus on hydrogen's LCA, especially in production. Osman *et al.* analyzed hydrogen's lifecycle—production, storage, and use—highlighting LCA's role in supporting a low-carbon future. Hammi and co-researcher investigated multiple hydrogen production pathways, such as biomass conversion, biogas reforming, thermochemical water splitting, and electrolysis, highlighting the significance of green hydrogen in supporting a carbon-neutral economy. In an additional research, Ang and Khoo evaluated different approaches to producing hydrogen using economic and ecological parameters.^161^ Their analysis indicated that while steam methane reforming and coal gasification present opportunities for improvement through carbon capture technologies or integration with renewable energy sources, methods like electrolysis and biomass gasification offer greater sustainability, albeit at a higher cost.^162^

**Fig. 22:**
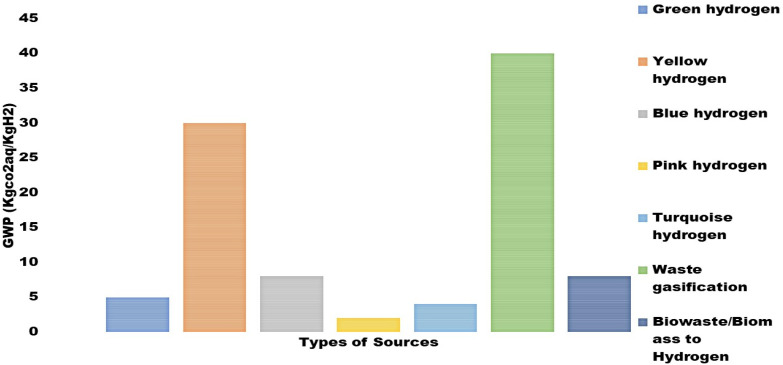
Comparative Analysis of the Global Warming Potential (GWP). Across various hydrogen production pathways.

Hydrogen produced through steam methane reforming generated an average of 11 kilograms of carbon dioxide per kilogram of hydrogen are shown in Fig. 22.^163^ In comparison, electrolysis powered by renewable energy and nuclear sources resulted in substantially lower emissions, at 2.02 and 0.41 kilograms of carbon dioxide per kilogram of hydrogen, respectively.^164^ However, electrolysis relying on grid electricity exhibited considerably higher emissions, reaching up to 41.4 kilograms of carbon dioxide per kilogram of hydrogen. Waste pyrolysis and gasification produced even greater emissions than steam methane reforming, whereas the use of residual biomass significantly reduced the associated carbon footprint. In terms of acidification potential, most methods showed comparable impacts, although biomass gasification was characterized by higher and more variable values.^165^

By integrating the performance characteristics of FCEVs with life cycle assessment data, a more accurate and complete understanding of their sustainability is achieved. For instance, while FCEVs offer zero emissions during operation, their true environmental value depends on whether the hydrogen used is derived from low-emission sources. In turn, life cycle data supports decisions on infrastructure investment, policy incentives, and fuel sourcing strategies.

This integrated approach ensures that hydrogen-powered mobility solutions align with climate targets and deliver measurable environmental benefits. It also supports ongoing innovation in vehicle technology, energy systems, and regulatory frameworks. Linking fuel cell vehicle development with life cycle assessment provides a powerful foundation for creating a cleaner, more efficient, and more responsible transportation future. Hydrogen derived from renewable sources offers a low-carbon energy pathway. Lifecycle assessments validate its environmental benefits compared to conventional fuels.

## 8. Harnessing hydrogen: innovations in energy and transport

### 8.1. Hydrogen fuel: challenges and opportunities for the future

Challenges and prospects for the hydrogen economy identifies major hurdles in hydrogen deployment such as cost, infrastructure, policy gaps, and offers outlooks on innovation and strategic pathways. Hydrogen's ability to reduce greenhouse gas emissions and improve performance has made it a popular sustainable fuel for the automobile and aviation industries.^166^ However, obstacles including exorbitant prices, inadequate infrastructure, and technological constraints prevent widespread implementation. Addressing these obstacles requires coordinated action from policymakers, industry leaders, and researchers. With the right support, hydrogen can move from niche applications to widespread use across sectors. Its environmental benefits make hydrogen especially attractive for decarbonizing transportation. Yet, storage, safety, and production costs remain significant concerns.^167^ Emissions can be reduced by producing hydrogen using sustainable energy sources such as hydropower, geothermal, solar, and wind. Legislators can assist by requiring quotas of renewable hydrogen and providing incentives for the construction of infrastructure. Industry collaboration on standards for storage and distribution can enhance safety and scalability. Research should focus on advanced storage materials and improved safety measures to ensure hydrogen remains a viable long-term solution.^168^

Hydrogen provides reduced CO_2_ emissions and better thermal efficiency in internal combustion engines (ICEs), but it can also result in pre-ignition in diesel engines and higher NO_*x*_ emissions.^169^ Optimizing combustion through injection timing, hydrogen blend ratios, and improved fuel-air mixing has shown promise, but further work is needed. Advancements in this area can make ICEs eco-friendlier and serve as a transitional path to hydrogen adoption using existing vehicle infrastructure.^170^ Policies supporting hydrogen-optimized ICEs—through tax credits or subsidies—can accelerate this shift while aligning with global sustainability goals.

In aviation, hydrogen use in gas turbines could significantly cut CO_2_ emissions. However, flame stability and NO_*x*_ emissions are technical challenges. New burner and injector designs are being developed to reduce emissions and improve efficiency.^171^ Success in aviation can influence other sectors like shipping and freight and promote investment in shared hydrogen infrastructure. International collaboration and funding are key to scaling hydrogen in high-energy-demand applications and setting global emission standards.^172^ Fuel cell electric vehicles (FCEVs) present another clean transportation option, producing only water vapor. Still, high costs, limited range, and sparse refueling networks hinder adoption. Ongoing R&D is targeting cost reductions and improved hydrogen storage, including on board production *via* ethanol steam reforming.^173^ As the technology matures, policy incentives and infrastructure investments can spur growth in the FCEV market and create jobs in the hydrogen sector. Integration of FCEVs with renewable hydrogen systems will also influence broader clean energy policies. To support hydrogen's growth, policymakers should implement financial incentives, fund infrastructure, and establish clear standards. Industry must invest in innovation and work across sectors to scale hydrogen technologies. Researchers should focus on lifecycle assessments, hybrid fuel systems, and practical infrastructure solutions. Regional studies can guide tailored strategies to enable widespread, efficient hydrogen integration into various energy sectors.^174^

### 8.2. Regulations and policy landscape in hydrogen fuel adoption

The acceleration of hydrogen integration into the energy landscape depends heavily on robust regulatory frameworks and the implementation of supportive policy measures. Japan provides a notable example, having revised its Hydrogen Basic Strategy in 2023 to strengthen its contribution to global climate initiatives. This update builds upon the foundation laid by its original 2017 strategy, which was the first comprehensive national roadmap for hydrogen deployment. The revised plan includes subsidies, incentives for renewable hydrogen, and targets such as 15 trillion yen in investments and a six-fold increase in hydrogen use by 2040. It also sets goals like producing 12 million tons of hydrogen annually, capping emissions at 3.4 kg CO_2_ per kg H_2_, and capturing 10% of the global electrolyzer market. Similarly, Germany's National Hydrogen Strategy promotes climate-friendly hydrogen through significant federal and state funding. It targets climate neutrality by 2045 and includes a detailed action plan to boost private investment. Germany expects hydrogen demand to hit 95–130 TWh by 2030, with 50–70% from imports, growing to 360–500 TWh by 2045, plus 200 TWh for derivatives.

Earlier hydrogen policies focused on R&D with little infrastructure planning, limiting adoption. Today, countries like Japan and Germany emphasize infrastructure development, refueling networks, and performance-based incentives. This shift reflects a broader strategy focused on coordinated policies and stakeholder collaboration to build sustainable hydrogen ecosystems. Table 3 compares existing and new policies and initiatives that facilitate the integration of hydrogen technology.

**Table 3:** Shows global hydrogen policies and strategies: a comparative analysis

S. No	Country/region	Policy/strategy	Year	Description	[Ref]
1	European Union	EU hydrogen strategy	2020	Focuses on green hydrogen production using renewable energy, aiming to install at least 40 GW of electrolyzers in the EU by 2030	175
2	Germany	National hydrogen strategy	2020	€9 billion investment to support green hydrogen production, infrastructure, and international partnerships	176
3	Japan	Basic hydrogen strategy	2017	First national hydrogen strategy; aims for a “hydrogen society” with focus on hydrogen power generation, fuel cell vehicles, and imports	177
4	South Korea	Hydrogen economy roadmap	2019	Aims to become a global leader in hydrogen by 2040, with millions of fuel cell vehicles and extensive infrastructure	178
5	Australia	National hydrogen strategy	2019	Focuses on becoming a major global hydrogen exporter, with support for both blue and green hydrogen technologies	179
6	China	Hydrogen industry development plan	2022	Targets 50 000 hydrogen fuel cell vehicles and a robust hydrogen production/supply system by 2025	180
7	United States	Hydrogen energy Earthshot (“hydrogen shot”)	2021	Aims to reduce the cost of clean hydrogen to $1 per 1 kg in 1 decade (“1 1 1′′ goal), with federal funding and R&D support	181
8	India	National green hydrogen mission	2023	Focuses on green hydrogen production with a target of 5 MMT (million metric tons) annually by 2030 and becoming a global hub for hydrogen exports	182
9	United Kingdom	UK hydrogen strategy	2021	Aims for 10 GW of low-carbon hydrogen production capacity by 2030, supporting both blue and green hydrogen pathways	183
10	Canada	Hydrogen strategy for Canada	2020	Targets net-zero emissions by 2050 with hydrogen playing a central role, emphasizing regional strengths and clean production methods	184

### 8.3. Hydrogen integration: lessons from industry case studies

The increasing relevance of hydrogen in energy systems is demonstrated by real-world instances. In order to support a national hydrogen strategy, the Japanese government has installed 180 hydrogen refueling stations and encouraged the use of fuel cell vehicles (FCVs), with the goal of selling 7600 FCVs by 2023.^185^ Companies like Toyota, Honda, Air Liquide Japan, ENEOS, and Iwatani are key players, while CJPT—a partnership of major automakers—is leading fuel cell truck projects, including a demonstration in Fukushima.^186^ Japan is also piloting hydrogen-powered trains, planning to use hydrogen and ammonia in ships and aircraft, and developing Woven City—a hydrogen-powered smart city with integrated pipelines and backup FC generators.^187,188^

The state of California authorized a 1.4 billion-dollar proposal (2024–2028) to expand completely emission-free transportation infrastructure, such as EV chargers and hydrogen facilities. Initial funding of $95.2 million in 2024–2025 supports workforce training and light-to heavy-duty ZEV infrastructure.^189^ In 2025–2026, GGRF funds add $510 million for at-home charging, school bus upgrades, and truck infrastructure.^190^ Though most funding dips in 2026–2027, $130 million is set aside for port ZEVs. The plan peaks in 2027–2028, with $436 million for EV charging and clean transport. By the end, California expects to reach 250 000 light-duty charging stations.^191^

### 8.4 Real-world applications and demonstration projects

The integration of hydrogen technologies into real-world energy systems has progressed significantly, with various demonstration projects highlighting their potential across different sectors. In the transportation sector, Germany has been at the forefront of deploying hydrogen-powered trains. Alstom's Coradia iLint, the world's first hydrogen fuel cell passenger train, has been operational in Lower Saxony, offering a zero-emission alternative for non-electrified rail lines. Similarly, the automotive industry has seen advancements with the deployment of fuel cell electric vehicles (FCEVs). Hyundai's NEXO FCEV exemplifies the integration of hydrogen technology into consumer vehicles, with South Korea actively promoting hydrogen as an alternative fuel and setting ambitious targets for hydrogen vehicle adoption. In the energy sector, large-scale green hydrogen production facilities have been established to support renewable energy integration. The Fukushima Hydrogen Energy Research Field (FH2R) in Japan utilizes a 10 MW-class hydrogen production unit powered by renewable energy, aiming to establish low-cost, CO_2_-free hydrogen production technologies. Additionally, the North Sea region is witnessing the development of offshore wind power hubs integrated with hydrogen production. Studies indicate that such integration can enhance energy system flexibility and contribute significantly to Europe's decarbonization goals. These examples underscore the practical applications of hydrogen technologies in real-world settings, highlighting their potential in achieving sustainable energy and transportation systems.^192–194^

### 8.5 Novelty and timely importance of the present review

(1) This review contributes a novel and timely perspective by providing a comprehensive and multisectoral analysis of hydrogen's role in sustainable transportation, encompassing internal combustion engines, gas turbine propulsion systems, and fuel cell electric vehicles, while also addressing critical issues of storage, safety, emissions, lifecycle assessment, and technoeconomic feasibility. Unlike previous reviews that focus on single technologies or regional contexts, this work offers an integrative outlook that aligns with ongoing global transitions toward low carbon energy systems. The review is particularly relevant given the increasing momentum in international hydrogen strategies such as the United States Department of Energy's Hydrogen Shot, the European Union's REPowerEU initiative, and ongoing hydrogen demonstrations in aviation, rail, and heavy transport sectors. Moreover, this review identifies several underexplored but rapidly advancing directions that hold promise for accelerating hydrogen deployment. These include the application of artificial intelligence and digital twin technologies to optimize hydrogen combustion, storage systems, and vehicle diagnostics; integration of hydrogen energy with smart grids and vehicle to grid applications through sector coupling; the development of circular economy approaches for end of life recovery of fuel cell and electrolyser components; and the need for equitable hydrogen deployment strategies in the Global South, particularly in emerging export regions facing water scarcity and infrastructure limitations. Additionally, recent progress in alternative hydrogen carriers such as ammonia, formic acid, and liquid organic hydrogen carriers offers promising solutions to overcome challenges associated with storage and long distance transport. By synthesizing these interdisciplinary aspects, the present review aims to consolidate existing knowledge while also guiding future research, policy development, and industrial practices in the expanding field of hydrogen-based transportation. Major barriers include infrastructure gaps, cost, safety, and regulatory alignment. Long-term success depends on coordinated global policies, technological advances, and market incentives.

**Fig. 23:**
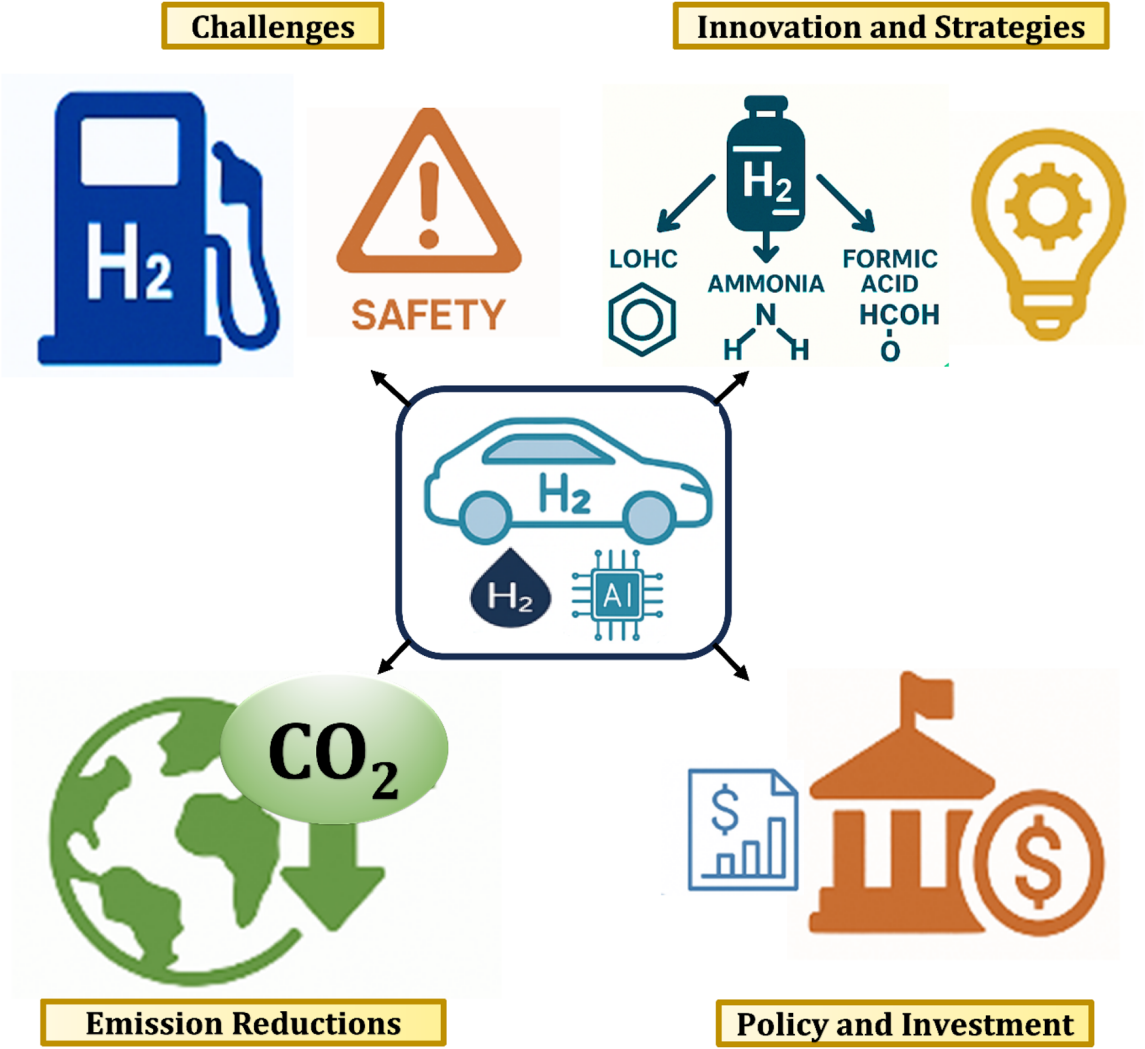
A schematic overview of future prospects in hydrogen energy, highlighting alternative carriers (LOHC, ammonia, formic acid), storage advances, policy support, and sustainable production pathways.

A schematic representation illustrating the promising prospects for the future of hydrogen energy transition was shown in Fig. 23. Key focus areas include alternative hydrogen carriers such as LOHC, ammonia, and formic acid; advancements in storage and distribution infrastructure; supportive policy frameworks and strategic investments; and sustainable hydrogen production pathways. These integrated approaches are essential for enabling a resilient and clean hydrogen-based energy future.

## 9. Conclusion

This review offers an in-depth evaluation of the prospective use of hydrogen as a viable energy transmitter across a range of applications, including internal combustion engines, fuel cell electric vehicles, and gas turbines. With the global emphasis increasingly directed toward cleaner energy alternatives to address climate transformation and decrease dependence on limited fossil fuel reserves, hydrogen emerges as a promising substance owing to its substantial amount of energy as well as its harmless combustion contaminants, primarily water vapour. However, the widespread adoption of hydrogen power sectors faces several technical and infrastructural challenges. In internal combustion engines, significant modifications are necessary to accommodate hydrogen safely and efficiently. Additionally, economic constraints and the absence of widespread infrastructure, such as refueling networks and distribution systems, remain significant barriers that must be overcome to facilitate broader adoption. Nevertheless, momentum is growing. Several major automotive manufacturers have already introduced hydrogen-powered vehicles to the market, and there is currently increasing curiosity about hydrogen as a prospective aviation fuel. Realizing the efficacy of hydrogen will require strong policy support, continued technological innovation, and cross-sector collaboration to overcome existing limitations. To pave the way for a hydrogen-driven future, future research should prioritize high-impact areas. Key areas for advancement include enhancing the efficiency of hydrogen production through innovative approaches such as electrolysis and photoelectrochemical systems, lowering production costs by developing affordable and widely available catalysts, and enlightening the competence and integration of the perfect hydrogen synthesis and supply chain. Equally important is the development of scalable distribution and storage systems, such as innovative materials for storing hydrogen and robust refuelling infrastructure. Assessing the practicality of hydrogen in the transportation sector requires a comprehensive and long-term perspective that accounts for technological advancements, economic feasibility, environmental impact, and infrastructure development. With coordinated global efforts, hydrogen can become a cornerstone of sustainable transport, enabling significant environmental, economic, and energy resilience benefits.

• Hydrogen enables deep decarbonization across multiple transport modes.

• It supports global climate and air quality goals through clean combustion.

• Technological innovation is crucial in storage, safety, and engine integration.

• Infrastructure development and regulation standardization are needed.

• Multinational policy coordination and funding can accelerate adoption.

• A hydrogen-driven economy promises both environmental and economic gains.

A comprehensive approach that incorporates environmental, social, economic, regulatory, and technical considerations is essential for guiding strategic planning and policy development. In conclusion, the incorporation of hydrogen into existing energy systems represents a pivotal step toward meeting global sustainability goals. With strong policy support and collaborative efforts from industry stakeholders, hydrogen has the potential to play a central role in the transition toward a cleaner and more resilient energy future, especially within the transportation sector.

## Conflicts of interest

There are no conflicts of interest to declare.

## Data Availability

No primary research results, software or code have been included and no new data were generated or analysed as part of this review.
